# Spatiotemporal evolutionary process of osteosarcoma immune microenvironment remodeling and C1QBP‐driven drug resistance deciphered through single‐cell multi‐dimensional analysis

**DOI:** 10.1002/btm2.10654

**Published:** 2024-04-10

**Authors:** Xin Wu, Ning Tang, Qiangqiang Zhao, Jianbin Xiong

**Affiliations:** ^1^ Department of Spine Surgery, Third Xiangya Hospital Central South University Changsha Hunan China; ^2^ Department of Orthopaedics, Third Xiangya Hospital Central South University Changsha Hunan China; ^3^ Department of Hematology Liuzhou People's Hospital affiliated to Guangxi Medical University Liuzhou Guangxi China; ^4^ Department of Hematology The Qinghai Provincial People's Hospital Xining Qinghai China; ^5^ Department of Orthopaedics Liuzhou Municipal Liutie Central Hospital Liuzhou Guangxi China

**Keywords:** C1QBP, drug resistance, macrophage polarization, osteosarcoma, tumor immune microenvironment

## Abstract

The tumor immune microenvironment has manifested a crucial correlation with tumor occurrence, development, recurrence, and metastasis. To explore the mechanisms intrinsic to osteosarcoma (OS) initiation and progression, this study synthesizes multiple single‐cell RNA sequencing data sets, constructing a comprehensive landscape of the OS microenvironment. Integrating single‐cell RNA sequencing with bulk RNA sequencing data has enabled the identification of a significant correlation between heightened expression of the fatty acid metabolism‐associated gene (*C1QBP*) and patient survival in OS. C1QBP not only amplifies the proliferation, migration, invasion, and anti‐apoptotic properties of OS but also instigates cisplatin resistance. Subsequent investigations suggest that C1QBP potentially promotes macrophage polarization from monocytes/macrophages toward M2 and M3 phenotypes. Consequently, C1QBP may emerge as a novel target for modulating OS progression and resistance therapy.

**Translational Impact Statement**

This study uncovers a pivotal link between the *C1QBP* gene and osteosarcoma (OS) progression, revealing its role in enhancing tumor growth, invasion, and drug resistance. By illustrating how C1QBP influences macrophage polarization in the tumor microenvironment, our findings offer a promising new target for OS treatment strategies, potentially improving patient outcomes and combating drug resistance. This breakthrough has significant implications for clinical interventions in OS, paving the way for more effective therapies.

## 1 INTRODUCTION

Osteosarcoma (OS), a principal type of primary malignancy originating in osseous tissue, exhibits its highest incidence rates among both adolescents and individuals aged over 60 years.^1^ Notably, the estimated annual incidence within pediatric and adolescent populations has escalated to between 3 and 4.5 cases per million.^2^ Patients presenting with metastatic disease typically demonstrate an event‐free survival rate subsuming 20%,^3^, ^4^, ^5^ and those diagnosed with unresectable primary or metastatic disease, or experiencing disease relapse, are confronted with notably adverse prognoses.^1^, ^6^ Recent years have witnessed an intensifying focus on the strong correlation between OS progress and the emergence of chemotherapy resistance.^7^, ^8^ Nevertheless, introducing novel therapeutic interventions for OS into clinical settings remains challenging, primarily due to its infrequent occurrence and marked tumor heterogeneity.^9^

The tumor immune microenvironment (TIME), encapsulating the immune constituents within tumors, has consistently illustrated a substantial association with tumor development and metastasis.^10^, ^11^ Predominantly, tumor‐associated M2 subtype macrophages among immune cells in TIME, orchestrate various processes, including extracellular matrix remodeling, cancer cell proliferation, metastasis, immunosuppression, and resistance to chemotherapeutic agents, significantly influencing both tumor development and responses to immunotherapy.^12^, ^13^ The metabolically hostile tumor microenvironment poses impediments to tumor‐infiltrating immune cells, obstructing the realization of enduring clinical remission with immunotherapy.^14^ An accumulating body of research accentuates the crucial role of metabolic reprogramming in macrophage activation, especially highlighting the imperative of enhanced fatty acid metabolism (FAM) for macrophage differentiation and activation.^15^, ^16^, ^17^

Modifications in FAM can amplify the migration and invasion of tumor cells by altering cellular signaling and managing epigenetic modifiers, which encompasses managing fatty acid homeostasis in relation to redox stress, averting ferroptosis, modulating membrane properties, and manipulating N^6^‐methyladenosine (m^6^A) to boost cancer motility and metastasis.^18^, ^19^, ^20^, ^21^, ^22^, ^23^ In OS, elevated FAM fosters tumor proliferation and invasion.^24^, ^25^, ^26^ However, the relationship between FAM and macrophages within the OS immune microenvironment remains to be elucidated. This study amalgamates single‐cell and bulk RNA‐seq data to anatomize the OS immune microenvironment and the role of macrophages, scrutinizing their association with FAM with the aim to elucidate the intricate interaction among these factors and offer insights into the mechanisms of OS progression, potentially unveiling new therapeutic strategies.

## 2 METHODS AND MATERIALS

### 2.1 Data collection

**FIGURE 1:**
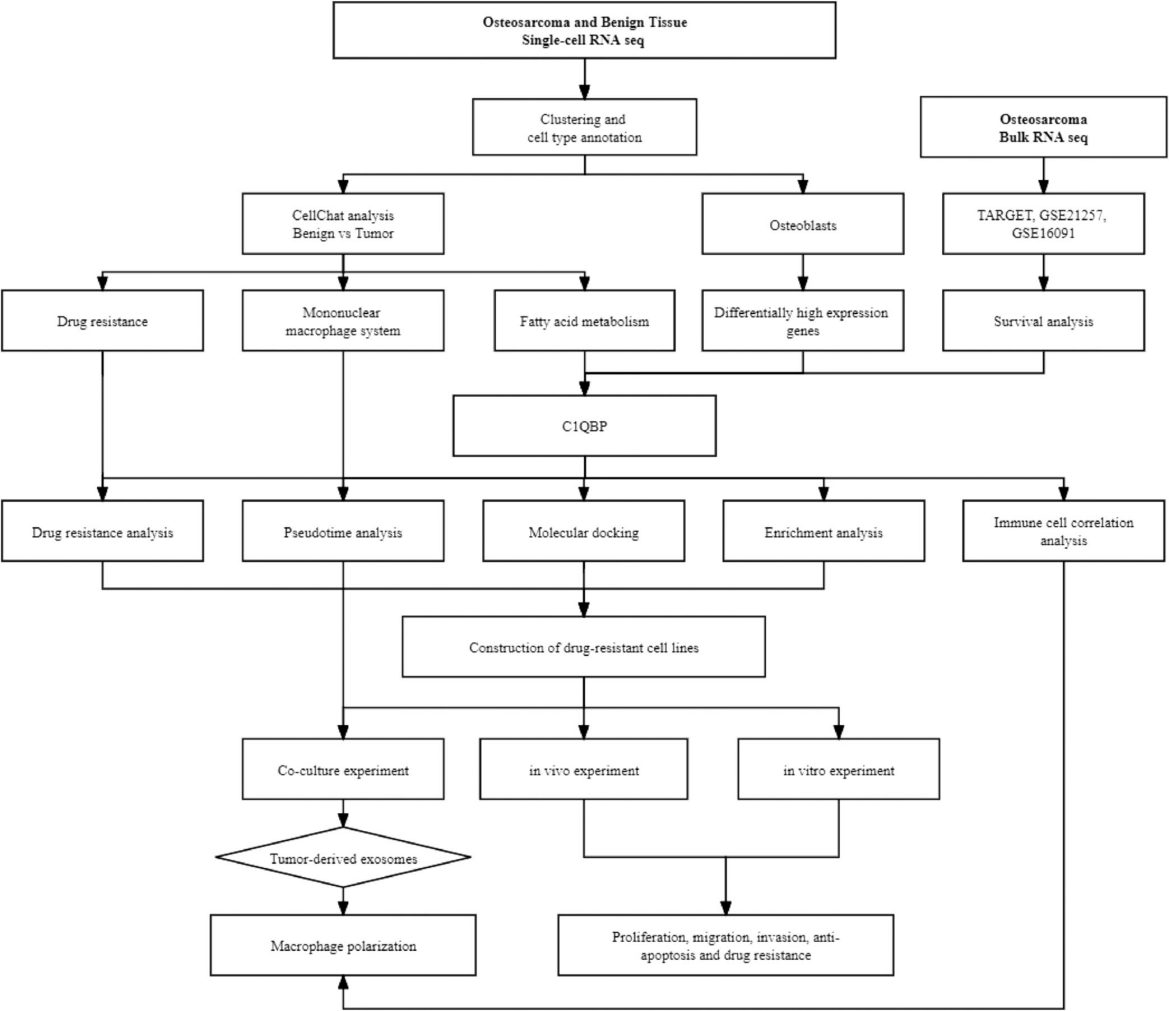
Flowchart of multidimensional data analysis in osteosarcoma research.

Single‐cell data were derived from several sources, including GSE152048,^27^ GSE162454,^28^ GSE169396,^29^ and GSE217792.^30^ The GSE152048 data set includes single‐cell data from 11 OS patients; the GSE162454 data set encompasses single‐cell data from six OS patients; and both the GSE169396 and GSE217792 databases contain single‐cell data from four individuals with control bone samples. The clinical details of the patients were extracted from the respective supplementary data in Data S1. Bulk RNA‐seq analyses, specifically from TARGET‐OS, GSE16091,^31^ and GSE21257,^32^ were procured from the TARGET program and the Gene Expression Omnibus (GEO) database. After filtering out patients with inadequate prognosis details, the GSE16091 data set comprises bulk RNA data for 34 patients with OS. The GSE21257 data set encompasses comprehensive data from 53 patients diagnosed with OS. Additionally, the TARGET database contains detailed prognosis data for 83 individuals afflicted with OS. The detailed information of all processes in this study is presented in the flowchart (Figure 1).

### 2.2 Single‐cell RNA‐sequence (scRNA‐seq) analysis

A total of 25 scRNA‐seq data sets were analyzed using the “Seurat” R package (version 4.3.0). Low‐quality cells were removed based on UMI count, nfeature, and mitochondrial gene thresholds to ensure data quality. The “DoubleFinder” package (version 2.0.3) was employed to exclude potential doublets and dead cells. Data normalization, scaling, and principal component analysis (PCA) were performed using the “LogNormalize” function. The “FindNeighbors” and “FindClusters” functions classified cells into clusters for cell type discovery, and both Uniform Manifold Approximation and Projection (UMAP) and t‐distributed stochastic neighbor embedding (t‐SNE) were employed to reveal distinct clusters.

### 2.3 Gene enrichment analysis

Gene Ontology (GO) analysis, Kyoto Encyclopedia of Genes and Genomes (KEGG) analysis, and identification of genes with differential expression and enrichment analysis were conducted using the “clusterProfiler” (version 4.8.1) package. Gene Set Enrichment Analysis (GSEA) and Gene Set Variation Analysis (GSVA) were executed employing R packages “GSEABase” (version 1.62.0) and “gsva” (version 1.48.1), respectively.

### 2.4 Cellular communication

Cell–cell communication analysis utilized the “CellChat” package (version 1.6.1) in R.^33^ An initial CellChat object was generated via the “createCellChat” function, employing RNA expression and cell data. Subsequent analyses encompassed ligand‐receptor interaction databases, which included categories such as “Secreted Signaling.”

### 2.5 Pseudotime analysis

This study employed Monocle 3 (version 3.1.0) for the analysis of single‐cell RNA sequencing data, a software package specifically designed for single‐cell transcriptomic analysis. Monocle 3 primarily aims to elucidate cell differentiation processes and the temporal dynamics of gene expression. The analysis commenced with the importation of scRNA‐seq data into a CellDataSet object created by Monocle 3. Following this, a range of preprocessing activities were conducted, including gene expression normalization, data quality assessment, and selective filtering of cells and genes. To streamline complex data sets and mitigate data noise, PCA was utilized for dimensionality reduction. Advanced techniques in Monocle 3, such as UMAP, were then applied for the spatial mapping of cell samples, aiding in the elucidation of cellular interactions and the spatial distribution of expression patterns. Cell differentiation pathways were inferred using Monocle 3's reverse graph embedding approach, enabling the construction of a cell state graph that vividly illustrates the dynamics of cell differentiation and pivotal transition points. The analysis of differentially expressed genes entailed contrasting gene expression variances across distinct cell states or pathway nodes. In conclusion, a variety of data visualization tools from Monocle 3 facilitated the detailed depiction of cell differentiation trajectories, differentially expressed genes, and gene expression patterns.

### 2.6 Determination and survival analysis of FAM‐related genes

Metabolic analysis was conducted on osteoblasts within the single‐cell data, segregating them into high and low FAM groups. A differential gene analysis was performed between these two groups, identifying 103 genes with high expression as FAM‐associated genes. For single‐gene survival analysis, we harnessed bulk RNA‐seq data sets, specifically focusing on the TARGET‐OS data set, alongside GSE16091 and GSE21257 data sets. The “survival” package (version 3.5‐5) was utilized to conduct survival analysis, specifically employing the log‐rank test, to compare the high‐ and low‐expression levels of each FAM‐related genes.^34^

### 2.7 Evaluation of immune cell subtype distribution

CibersortX and mean expression of characteristic genes were used to elucidate cellular subtype infiltration across the TARGET clinical cohort. Spearman's correlation analysis uncovered associations between C1QBP and various cell clusters.

### 2.8 OncoPredict for drug sensitivity analysis

The OncoPredict package, an R tool used for drug response prediction,^35^ was utilized to investigate the relationship between the expression of C1QBP and sensitivity to commonly used chemotherapy and molecular targeted drugs. A comprehensive analysis was conducted on 198 drugs to assess their sensitivity differences between high‐risk and low‐risk groups, employing unpaired *t*‐tests as the analytical method. The significance threshold was established at *p* < 0.05.

### 2.9 Molecular docking analysis

Target protein crystal structures were acquired from SWISS‐MODEL and stored in “PDB” format files for analysis. Key drug structures were sourced from PubChem database in “sdf” format, and their conversion to mol2 format was facilitated using Open Babel GUI software (Version 3.1.1). PyMOL (Version 2.5.7) and AutoDock4 (Version 4.2.6) were employed for preprocessing and molecular docking experiments. The preprocessing steps involved the removal of water molecules and the addition of nonpolar hydrogen atoms to both the protein and drug molecules. Molecular docking was performed using AutoDock4. Subsequently, the results were visualized using PyMOL for a comprehensive understanding. Effective docking was determined based on the criterion that the binding energy values should be less than −2.0 kcal/mol.

### 2.10 Establishment of OS drug‐resistant cell lines

OS‐resistant cell lines, designated MG63/CDDP and U‐2OS/CDDP, were developed to exhibit cisplatin resistance, originating from their parental lines MG63 and U‐2OS, respectively. Parental cells were seeded into culture dishes and, upon reaching 60%–70% confluence, treated with cisplatin, starting at a concentration of 1 μM. After 48 h, the cisplatin‐containing medium was replaced with a complete medium, and cells were further cultured until optimal growth was attained. This procedure was reiterated six times per concentration, gradually amplifying cisplatin concentrations (1, 2, 4, 8, 16, and 32 μM), each concentration applied in six repetitions. After 9 months, OS cell lines resistant to cisplatin were successfully established.

### 2.11 Reverse transcription quantitative polymerase chain reaction (RT‐qPCR)

RNA was isolated using the GeneJET RNA Purification Kit (Thermo Fisher Scientific, USA) and reverse‐transcribed using the RevertAid First Strand cDNA Synthesis Kit (Thermo Fisher Scientific, USA). Subsequent qPCR analysis was performed using the SYBR Premix ExTaq (TaKaRa, Japan) on the Roche LightCycler 480 II system, with specific mRNA primer sequences provided in Table S1.

### 2.12 Short hairpin RNA (shRNA)

ShRNAs targeting human C1QBP are designated as shRNA1‐C1QBP, shRNA2‐C1QBP, and shRNA3‐C1QBP. Upon reaching 80% confluence, OS cells, seeded at 1 × 10^5^ cells/well in 6‐well plates, were transfected with shRNA using Lipofectamine™ 3000. mRNA levels were assessed via RT‐qPCR post‐transfection to determine shRNA efficacy, with the experiment conducted in triplicate.

### 2.13 CCK‐8 analysis

Cells were cultured in 96‐well plates with transparent bottoms and treated as described in the accompanying figure legend. Following the manufacturer's guidelines, cellular proliferation was assessed using the CCK‐8 assay kit (Sigma‐Aldrich, St. Louis, MO). Absorbance was measured at 450 nm, and readings were normalized by subtracting the reference wavelength's absorbance. Data are presented as relative optical density.

### 2.14 EdU staining procedure

The cells were incubated with 10 μM EdU labeling solution (Apexbio) for 2 h, following the manufacturer's protocol. Subsequently, 500 μL of Click reaction mixture containing Cy3 azide was added to each well and incubated in the dark at room temperature for 30 min. After washing with PBS, the cells were further incubated with 1 mL of Hoechst 33342 solution (final concentration 5 μg/mL) under the same conditions for an additional 30 min. Fluorescence microscopy was then employed for visualization.

### 2.15 Migration and invasion analysis

In this study, our focus was on two OS cell lines, MG63/CDDP and U2OS/CDDP, for which we conducted Transwell migration and invasion assays. These cell lines were cultured in a complete medium, enriched with 10% fetal bovine serum, and sustained at 37°C with 5% CO₂. The aim of our research was to investigate the effect of *C1QBP* gene silencing on cell migration and invasion capabilities. To achieve this, siRNA technology was employed to target and silence the *C1QBP* gene specifically. In the experimental procedure, cells subjected to treatment and their respective control groups were placed in the serum‐free upper chamber of a Transwell apparatus (Corning brand), with the lower chamber containing a medium supplemented with 10% FBS to serve as a chemical attractant. For invasion assays, the Transwell membrane's upper chamber was pre‐coated with Matrigel. Following a 24‐h incubation period, cells that failed to migrate were removed from the upper membrane, whereas those that migrated to the lower chamber were fixed in formaldehyde and stained with crystal violet. The specific impact of *C1QBP* gene silencing on the migration and invasion capacities of the MG63/CDDP and U2OS/CDDP cell lines was quantitatively determined through microscopic counting and analysis.

### 2.16 Caspase 3 activity

Caspase 3 activity was assessed utilizing the Beyotime Caspase 3 Activity Assay Kit. The procedure began by lysing the treated cells with the lysis buffer included in the kit, followed by centrifugation to separate the supernatant. The resulting cell lysate was then combined with Ac‐DEVD‐pNA at a concentration of 2 mmol/L and incubated at 37°C for 2 h. Post‐incubation, a microplate reader was employed to measure the absorbance at 405 nm, thereby determining the activity of Caspase 3.

### 2.17 Western blot analysis

Proteins were extracted using radioimmunoprecipitation assay buffer (Beyotime, China) and quantified with the BCA assay (Beyotime). Samples were separated using 10% SDS‐PAGE and transferred to polyvinylidene difluoride membranes (Sigma‐Aldrich, USA). Membranes were probed with primary antibodies, including anti‐C1QBP (1:1000, Cell Signaling Technology), anti‐BCL2 (1:200, Abcam), anti‐BAX (1:1000, Abcam), anti‐CD9 (1:1000, Cell Signaling Technology), anti‐Calnexin (1:500, Cell Signaling Technology), anti‐TSG101 (1:2000, Abcam), anti‐Calnexin (1:1000, Abcam), anti‐CD86 (1:1000, Abcam), anti‐INOS (1:1000, Cell Signaling Technology), anti‐CD206 (1:1000, Proteintech, China), anti‐ARG1 (1:1000, Proteintech), anti‐FABP4 (1:1000, Cell Signaling Technology), anti‐beta tubulin (1:5000, Proteintech), and anti‐GAPDH (1:10,000, Proteintech) followed by secondary antibody incubation. Signals were detected using a UVP ChemStudio System (Ultraviolet Products, USA) and analyzed with VisionWorks software (Analytik Jena, Germany).

### 2.18 Immunofluorescence analysis

Apoptosis was detected using TUNEL staining, and the expression of the *C1QBP* gene was assessed through immunofluorescence. Protocols were followed as per the manufacturers' instructions. Cellular nuclei were visualized using DAPI staining, and images were captured with a laser confocal microscope.

### 2.19 Flow cytometric assessment of cell apoptosis

Post‐transfection, culture supernatants were collected after 48 h. Cells were dissociated with trypsin without EDTA, creating single‐cell suspensions. The suspensions were centrifuged, washed, and stained with Annexin V‐FITC and propidium iodide. Cellular apoptosis was immediately assessed using flow cytometry.

### 2.20 Soft agar colony formation

A base layer of 0.6% low‐melting agarose was prepared in complete MEM medium. An upper layer with 0.3% agarose was then added, containing 5000 predetermined cells. Dishes were incubated at 37°C with 5% CO_2_ for about 2 weeks. Colonies were stained with a solution of 0.5% crystal violet and 25% methanol for 20 min. Dishes were scanned and colonies quantified using Quantity One v.4.0.3 software (Bio‐Rad, USA).

### 2.21 Isolation, characterization, and investigation of exosome

Exosome isolation and characterization methods are detailed in previous research.^36^ The medium was replaced with 10% exosome‐free FBS in DMEM. Supernatants were collected and centrifuged, and the final pellet was subjected to ultracentrifugation. For electron microscopy, a sample was placed on a copper grid, dried, and stained. Exosome morphology was examined using a JEM‐F200 transmission electron microscope (JEOL, Japan). Particle size was determined using a NanoSight NS500 (Malvern Pananalytical, USA). Exosome markers were identified via western blotting.

### 2.22 Animal experiment

Ethics approval was obtained by the Institutional Animal Care and Use Committee (IACUC) of Central South University (CSU‐2022‐0700). Female BALB/c nude mice, aged 6–8 weeks, were divided into two groups: shRNA‐NC (comprising underarm shRNA‐NC/MG63/CDDP cell inoculation and cisplatin intraperitoneal injection) and shRNA‐C1QBP (consisting of underarm shRNA‐C1QBP/MG63/CDDP cell inoculation and cisplatin intraperitoneal injection). Each group contained three mice. Tumor growth was monitored, and cisplatin was administered once tumors reached 50 mm^3^. After 4 weeks, mice were euthanized, and tumors were examined for C1QBP and TUNEL expression, as well as HE staining.

## 3 RESULTS

### 3.1 Comprehensive examination of OS tumor microenvironment at single‐cell resolution

**FIGURE 2:**
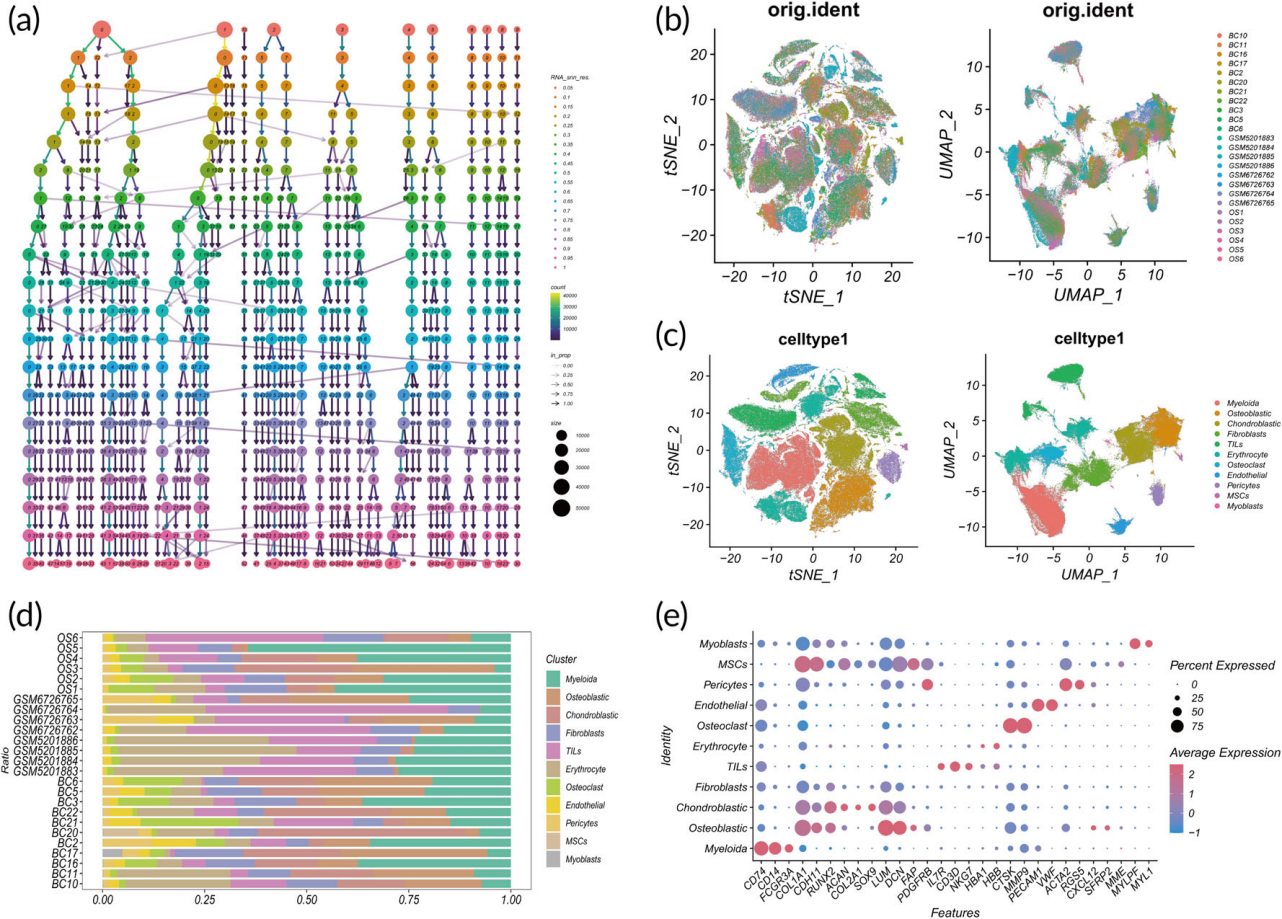
Comprehensive transcriptomic analysis of the osteosarcoma single‐cell RNA landscape. (a) Subclustering of all cells is illustrated at multiple resolutions. (b) t‐SNE and UMAP plots of 25 samples are depicted. (c) t‐SNE and UMAP plots present the 11 primarily identified cell types within osteosarcoma. (d) The relative distribution of each cell cluster is shown across 25 samples. (e) Dot plots, where dot size signifies the proportion of cells expressing specific markers and color intensity indicates average marker expression level, exhibit the expression of 29 marker genes across the 11 cell clusters. t‐SNE, t‐distributed stochastic neighbor embedding; UMAP, Uniform Manifold Approximation and Projection.

This study analyzes previously published single‐cell data sets (GSE152048, GSE162454, GSE169396, and GSE217792) to scrutinize the OS tumor microenvironment. After excluding cells with elevated mitochondrial and red blood cell gene expressions, we executed dimensionality reduction clustering using HVG analysis and PCA (Figure S1a,b). An assessment was conducted on the influence of cell cycle genes on cell clustering (Figure S1c), and correlation analyses among genes related to mitochondria, red blood cells, nfeature counts, and ncount (Figure S1d–f) were performed. A detailed exposition regarding nfeature, ncount, G2M scores, and S score for each patient is presented (Figure S1g–j). After stringent quality control and batch effect removal, a total of 194,374 cells were analyzed. We utilized clustree to exhibit the impact of different clustering resolutions on cell grouping through tree‐like diagrams (Figure 2a). Inspection of t‐SNE and UMAP plots after batch effect removal revealed a homogenous cell distribution, signifying the successful eradication of batch effect (Figure 2b). Employing marker genes from prior literature,^27^ 10 prominent cell clusters were identified (Figure 2c). Notably, substantial variations in the proportions of cell clusters among lesions signified both tumor heterogeneity and lesion consistency (Figure 2d). Dot plots facilitated a comparison of the proportion of cells expressing cluster‐specific markers and their relative expression levels (Figure 2e).

### 3.2 Osteoblastic cells demonstrate heterogeneity in OS and control bone

**FIGURE 3:**
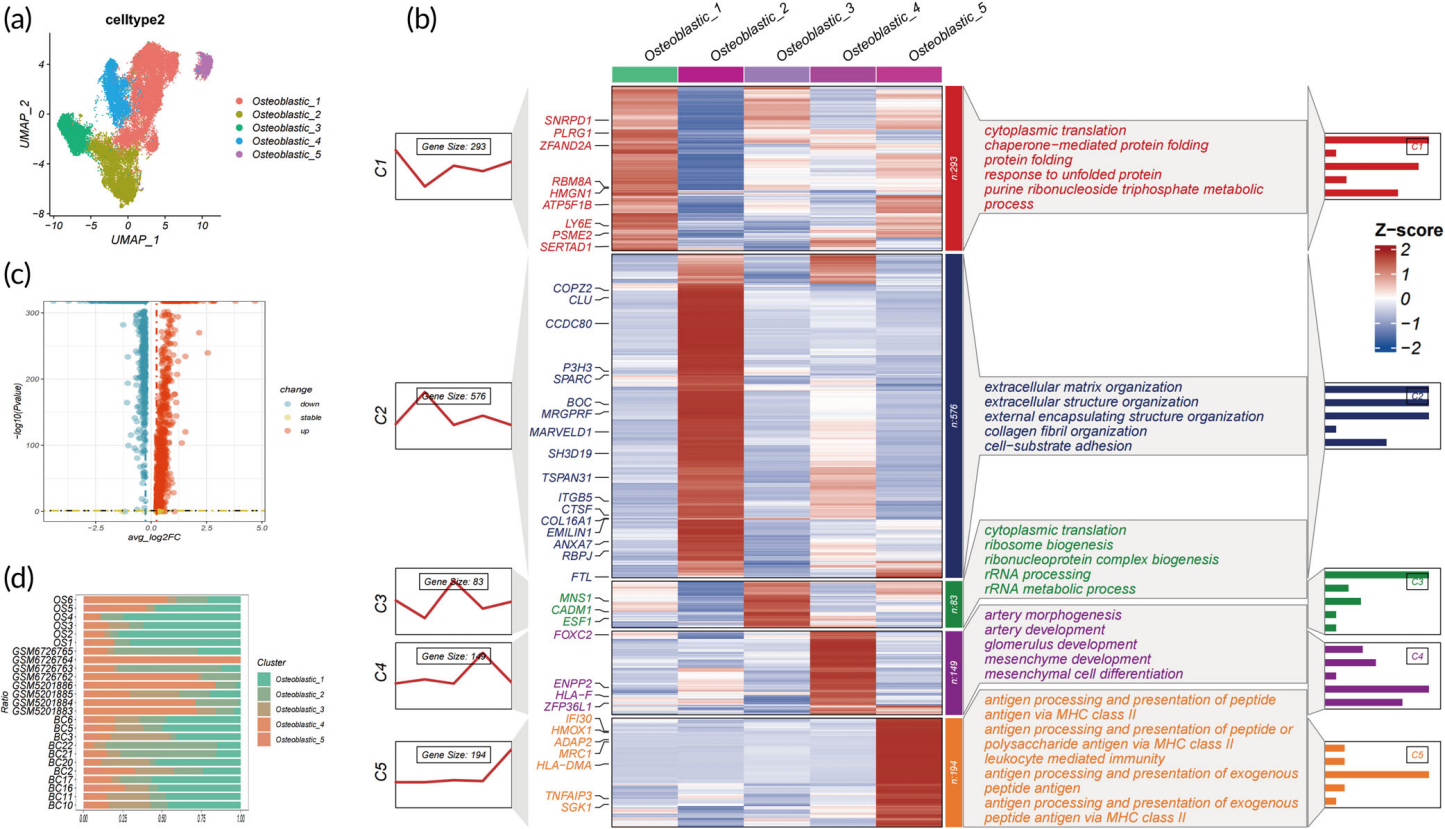
Osteosarcoma heterogeneity. (a) Identification of five principal malignant OS cell subtypes via UMAP analysis. (b) Expression heatmap portraying DEGs and performing GO analysis across five malignant osteosarcoma cell subclusters. (c) A volcano plot illustrating DEGs between control and osteosarcoma cells. (d) The relative proportions of each cell cluster across the 25 samples. GO, Gene Ontology; OS, osteosarcoma; UMAP, Uniform Manifold Approximation and Projection.

Osteoblastic cells emerge as the paramount subtype both in clinical OS and in control bone tissues. Employing UMAP analysis and marker gene identification, five osteoblastic lineages have been discerned (Figure 3a). These diverse subgroups of osteoblastic cells exhibit notably disparate transcriptomic profiles and pathway enrichments (Figure 3b). Specifically, Osteoblastic_1 predominantly enriches in processes related to cytoplasmic translation and chaperone‐mediated protein folding, suggesting a potential promotion of tumorigenesis through aberrant protein synthesis. Osteoblastic_2 is chiefly enriched in pathways associated with extracellular matrix and structure organization, hinting at a potential impact on tumor invasion and metastasis by modulating the extracellular matrix and structures. Osteoblastic_3 primarily exhibits enrichment in ribosome biogenesis and ribonucleoprotein complex biogenesis, indicating perturbations in cellular ribosomes and RNA processes. Osteoblastic_4 displays principal enrichment in pathways related to artery morphogenesis and mesenchymal cell differentiation, implying anomalous activities in the developmental processes of osteoblastic cells and potential interactions with various tissues or cell types. Lastly, Osteoblastic_5 demonstrates enrichment in antigen processing and presentation processes, revealing a possible association with immune system activity and antigen processing. Collectively, osteoblastic cells in OS present markedly distinct expression profiles compared to their control counterparts (Figure 3c), with notable variances in cell cluster distribution across lesions highlighting the heterogeneity within osteoblastic cells (Figure 3d).

### 3.3 Heterogeneity of myeloid cells in the immune microenvironment

Within the immunological microenvironment of OS, myeloid cells play multifaceted roles in malignancy progression. They not only possess the capability to foster tumor progression, invasion, and metastasis but also actively participate in regulating immune responses, thereby influencing treatment outcomes.^37^ To ascertain the optimal clustering resolution, illustrated in Figure S2a, we systematically evaluated the impact of various resolutions on cell clustering. Utilizing UMAP visualization, a two‐dimensional cell distribution was observed, which was characterized by an absence of discernible relationships among samples, indicating the successful mitigation of batch effects. Subsequently, six discrete myeloid cell subgroups were identified, namely M1_TAM, M2_TAM, Monocytes, M3_TAM, dendritic cells (DCs), and Neutrophils, with the chosen resolution accentuated during subgroup identification. M3_TAM is a cell type newly identified in the referenced OS single‐cell data, characterized by high expression of the *FABP4* gene, and other types of myeloid cells were identified according to classic markers.^27^ Predominantly, myeloid lineage cells are derived from malignant tumor tissue, although a minority are also present in control tissue (Figure S2b). Ridgeline plots and dot plots, showcasing previously investigated marker genes, underscored their efficacy in delineating cell subgroups (Figure S2c,d). Concomitantly, it became apparent that myeloid cells infiltrating the tumor microenvironment predominantly comprised M2_TAMs, DCs, and Neutrophils. Nonetheless, substantial disparities in cell proportion were observed across different samples, reflecting the tumor's heterogeneity and consistency (Figure S2e). Myeloid cells derived from tumor tissue exhibited significant transcriptomic expression differences when compared to those from control tissue (Figure S2f).

### 3.4 Subgrouping of tumor‐infiltrating lymphocytes (TILs)

**FIGURE 4:**
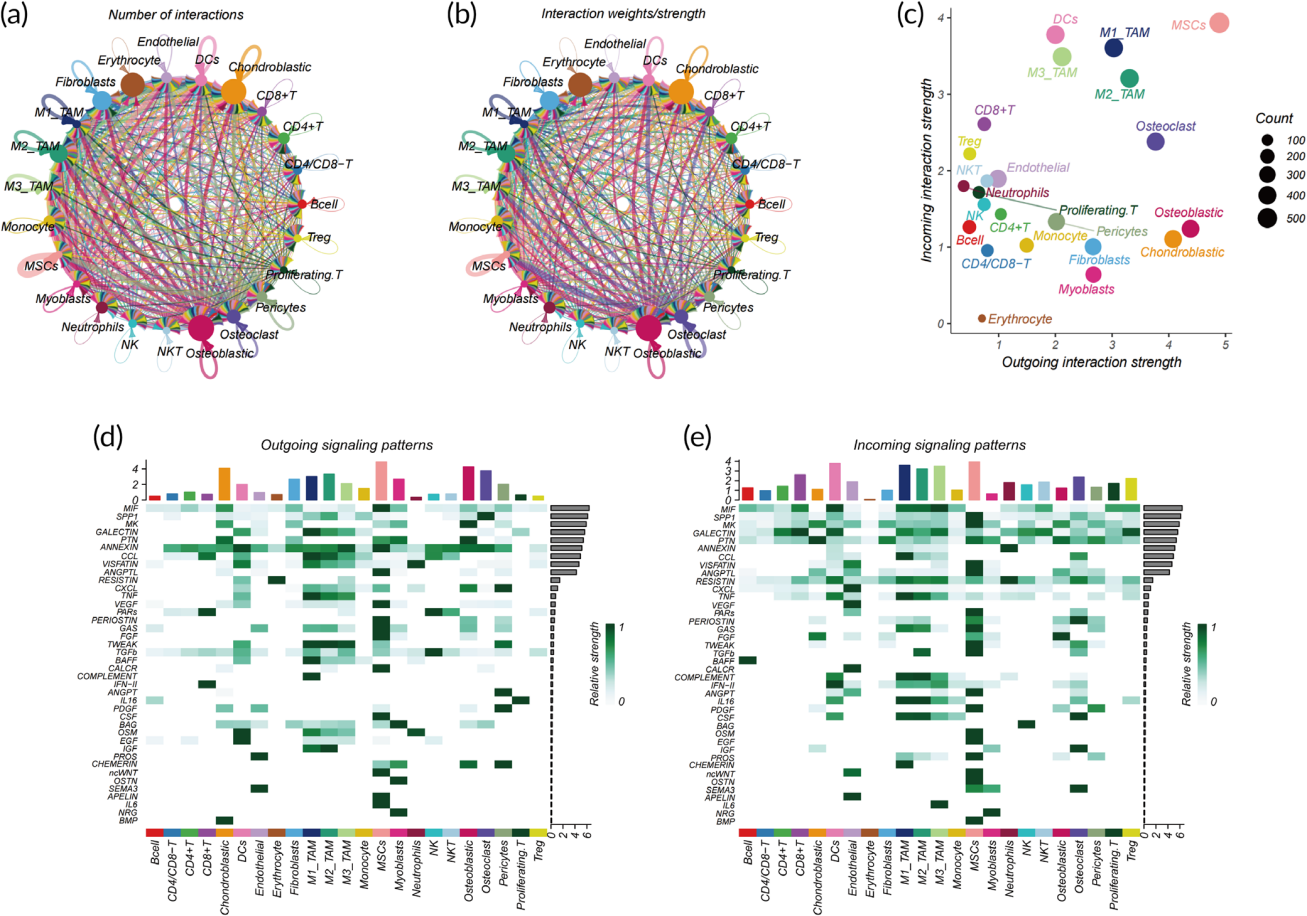
Extensive intercommunication among cellular subpopulations. (a) Communication between cellular subpopulations quantified by intensity and (b) quantity. (c) Two‐dimensional visualization of dominant emitter (source) and receiver (target) cells. (d) Predominant outgoing signaling patterns. (e) Predominant inbound signaling patterns.

TILs constitute a distinct subset of immune cells within the tumor microenvironment and have frequently demonstrated substantive associations with immune surveillance and prognostic assessments. A plethora of research has robustly evidenced the capacity of TILs to act as predictive markers for immunotherapeutic responses.^38^, ^39^, ^40^ Our identification revealed eight distinct cellular subgroups, namely CD8+ T cells, CD4+ T cells, CD4/8‐T cells, NK cells, B cells, T‐regulatory cells (T‐reg), proliferating T cells, and additional NKT cells (Figure S3a). UMAP heatmaps and bubble plots visualization elucidates that marker genes exhibit outstanding discriminative capabilities in distinguishing these cellular populations (Figure S3b and Figure 4c). The gene expression patterns and their associated biological functions notably diverge among various TIL subtypes (Figure S3d). For instance, CD8+ T cells, which predominantly enrich in the T cell receptor signaling pathway and leukocyte cell–cell adhesion, play a pivotal role in modulating the immune system and orchestrating cellular interactions. CD4+ T cells, crucial for protein synthesis and ribosomal function, primarily involve pathways related to cytoplasmic translation and ribosome‐associated processes. Similarly, CD4/8‐T cells, being significantly enriched in oxidative phosphorylation and purine metabolism, potentially play an integral role in cellular energy production and related biological processes. Other cells, such as NK and B cells, demonstrate primary enrichment in processes related to their respective immune functions, while T‐reg and NKT cells underscore significant roles in intracellular protein degradation and antiviral responses, respectively. Despite the expression of TILs in all patients, significant disparities in the distribution of cellular subgroups were observed, underscoring a pronounced heterogeneity within TILs, as demonstrated in Figure S3e.

### 3.5 Cellular communication within the OS‐TIME

Within the TIME, intercellular communication significantly influences cancer development and treatment outcomes.^41^ This communication encompasses a myriad of factors such as immune responses, vascular supply, tissue architecture, and cellular heterogeneity. Collectively, these factors dictate tumor growth, invasion, metastasis, and therapeutic efficacy.^42^ Our study revealed pronounced richness and intensity in the intercellular communication among distinct cellular subgroups (Figure 4a,b, Figures S4 and S5). Visual analysis indicated that osteoblastic cells, chondroblastic cells, and osteoclasts predominantly function as the primary sender cells, while M1_macrophages, M3_macrophages, M2_macrophages, and DCs primarily act as receiver cells (Figure 4c). Bone marrow stromal cells serve dual roles, both as the primary recipient and the primary sender (Figure 4c). Furthermore, osteoblastic and chondroblastic cells exhibit the capability to influence various cellular subgroups through a range of signaling pathways (Figure S6). A deeper exploration into the distribution of outgoing and incoming signals among these subgroups pinpointed pivotal signaling molecules, such as MIF, SPP1, MK, GALECTIN, and others, as central contributors (Figure 4d,e).

### 3.6 Coordinated communication within the OS‐TIME

**FIGURE 5:**
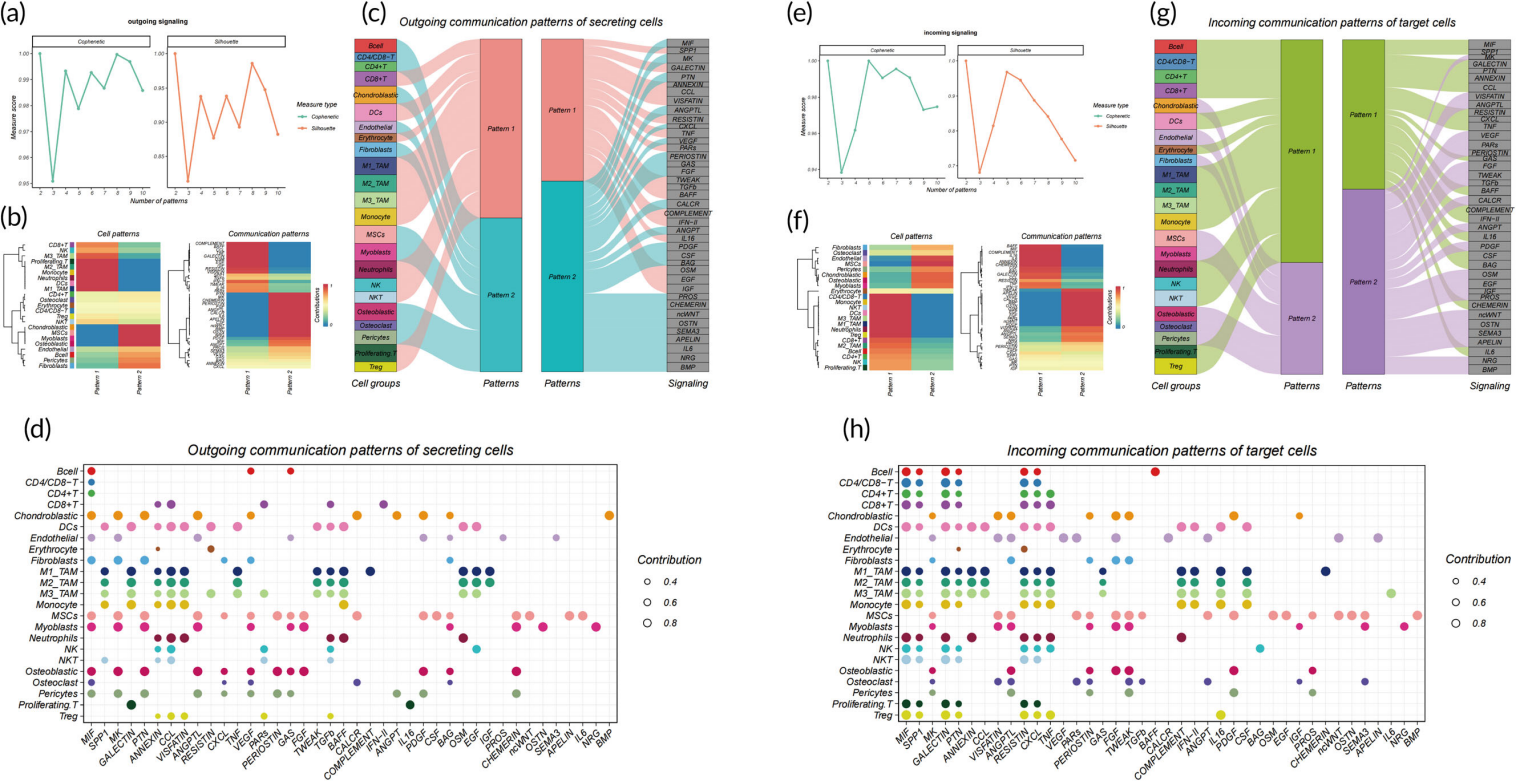
Systematic analysis of global cell communication patterns. (a) Inference of output pattern numbers via cophenetic and silhouette values. (b) Identification of cell subpopulations and corresponding signal patterns for output. (c) Sankey plot visualization of outgoing signal patterns from secreting cells. (d) Variation plot detailing outgoing signals in vital pathways. (e) Estimation of input pattern numbers via cophenetic and silhouette values. (f) Identification of cell subpopulations and corresponding signal patterns for input. (g) Sankey plot visualization of inbound signal patterns for receiving cells. (h) Variation plot detailing incoming signals in essential pathways.

We leveraged the predictive capabilities of CellChat to discern coordinated cellular responses via pattern recognition in our study. Utilizing this tool, we unveiled predominant communication patterns among cells by adopting a robust pattern recognition methodology. Employing selectK, we quantified both output and input patterns (Figure 5a,e), subsequently categorizing cell subtypes and communication patterns into discrete categories (output, *n* = 2; input, *n* = 2) (Figure 5b,f). Moreover, we graphically illustrated the inferred potential output patterns and their associations with cellular populations and signaling pathways, utilizing Sankey diagrams (Figure 5c,g). Additionally, a thorough pattern recognition analysis was conducted to explore the subtle variations in outgoing and incoming signals across all significant pathways (Figure 5d,h). Our findings underscore the critical roles of osteoblastic cells, M1 macrophages, M2 macrophages, and M3 macrophages within the overarching communication framework.

### 3.7 Discrepancies in cellular communication between OS and control bone

**FIGURE 6:**
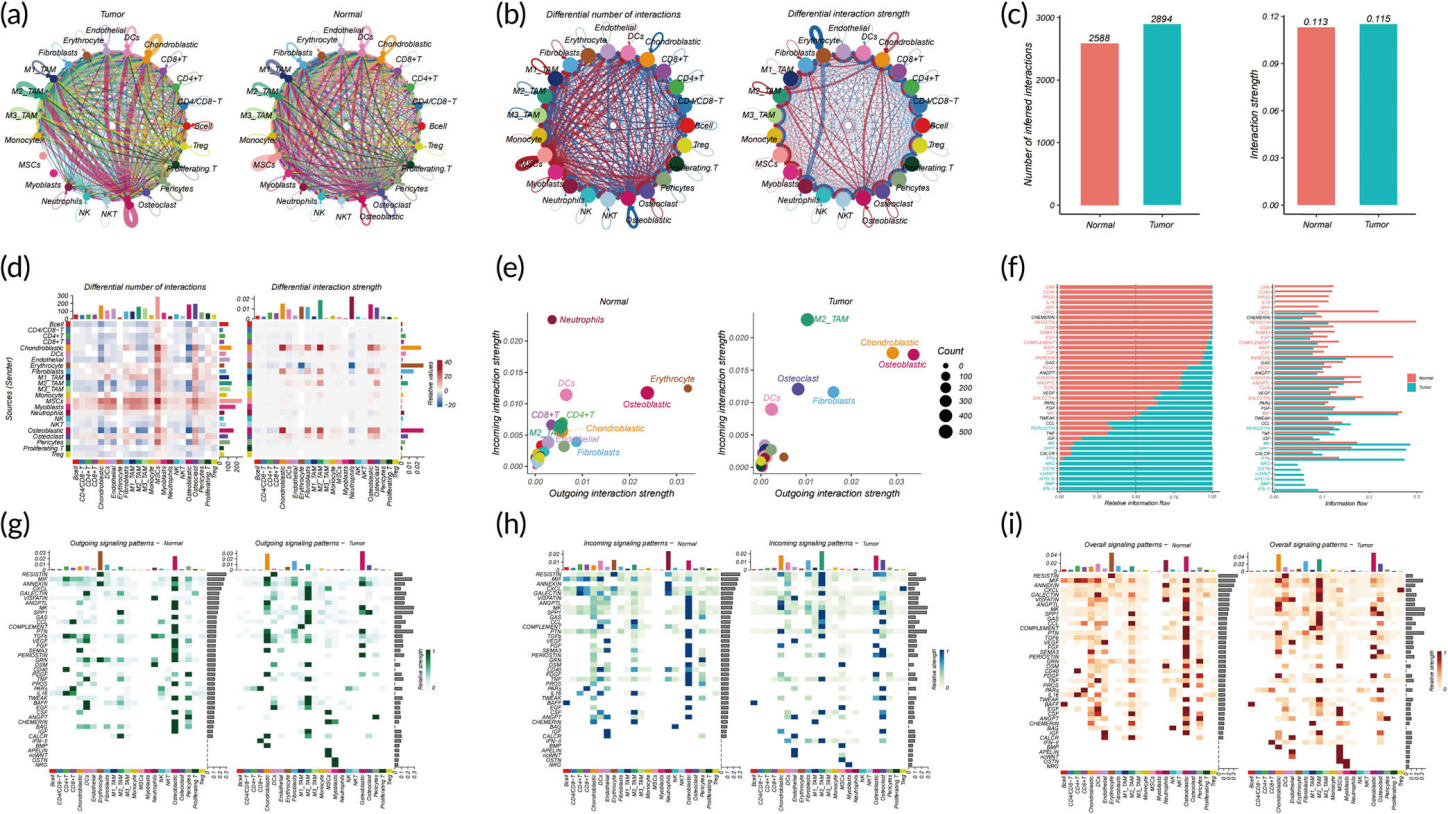
Comparative analysis of cell communication: osteosarcoma versus control bone tissues. (a) Quantification of cell interactions between osteosarcoma and control bone tissues. (b) Differential expression of interaction quantity/intensity in osteosarcoma (red and blue lines represent high and low expression, respectively). (c) Interaction quantity/intensity comparison between osteosarcoma and control bone tissues. (d) Heatmap of differential interaction quantity/intensity. (e) Comparison of primary sources and targets in a two‐dimensional spatial context. (f) Comparative analysis of overall information flow in signaling pathways. (g and h) Comparison of outgoing (g) and incoming (h) signals for each cell subpopulation. (i) Comparative analysis of overall signaling for each cell subpopulation.

OS and control bone tissues manifest complex cellular communication networks (Figure 6a), displaying discernible disparities in interaction quantity and intensity (Figure 6b). OS distinctly exhibits an augmentation in both overall interactions and interaction intensity relative to control bone tissue (Figure 6c). Through the use of heatmaps and two‐dimensional (2D) plots, we elucidate differences in cellular communication among cell subtypes. Our analyses uncover that osteoblastic cells serve pivotal roles as both senders and receivers in both control bone and OS, whereas M2 macrophages demonstrate markedly enhanced receiving capabilities in comparison to control bone tissue (Figure 6d,e). To delve deeper into the mechanisms underlying OS recurrence, we examine disparities in signaling pathways among cell subtypes. Contrary to control bone tissue, specific pathways (e.g., IFN‐II, BMP, APELIN, ncWNT, OSTN, NRG) manifest significant overexpression in OS (Figure 6f). Noteworthy is that these highly expressed pathways robustly correlate with FAM, the mononuclear phagocyte system, and the incidence and progression of cancer, along with drug resistance.^43^, ^44^, ^45^, ^46^, ^47^, ^48^, ^49^, ^50^, ^51^ Furthermore, our comparative analysis of specific signaling pathways and ligand receptors, which regulate communication probabilities between primary OS and control bone tissue, elucidates distinctions in incoming and outgoing signals, as well as overall communication probabilities between OS and control bone tissue (Figure 6g–i, Figure S7a–c).

### 3.8 The regulatory implications of FAM‐related gene *C1QBP* in OS

**FIGURE 7:**
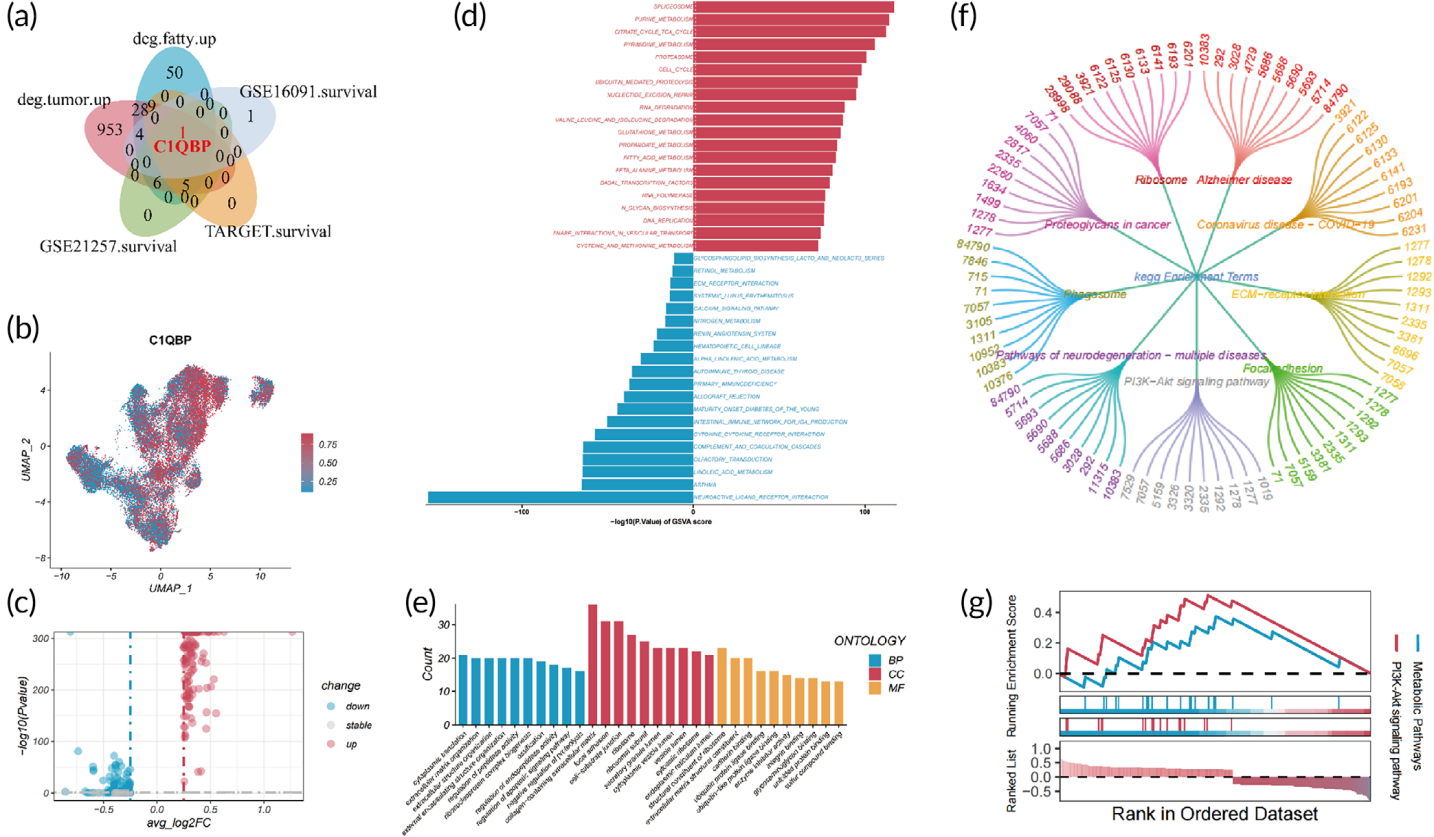
Significance of C1QBP regulation in osteosarcoma. (a) Venn diagram illustrating the intersection of fatty acid metabolism (FAM) and highly expressed genes in osteosarcoma (OS), and prognostic genes. (b) UMAP plot of C1QBP expression. (c) Volcano plot of genes differentially expressed relative to C1QBP expression levels. (d–g) Analyses via GSVA, GO, KEGG, and GSEA of DEGs. GO, Gene Ontology; GSEA, Gene Set Enrichment Analysis; GSVA, Gene Set Variation Analysis; KEGG, Kyoto Encyclopedia of Genes and Genomes; UMAP, Uniform Manifold Approximation and Projection.

Upon isolating osteoblasts from both control and tumor tissues utilizing a single‐cell UMAP plot, we observed distinct expression patterns between the tissue types (Figure S8a,b). Additionally, depicting the expression profiles of FAM‐related genes within the same plot revealed notable disparities between tumor and control tissues (Figure S8c,d). Our intersection analysis, encompassing 103 genes related to FAM and 1006 genes exhibiting significant differential expression in osteogenic OS cells, identified 53 OS‐related FAM genes (OS‐FAMs) (Figure 7a). Then, validation of survival outcomes associated with OS‐FAMs, using the GSE21257, GSE16091, and TARGET OS public databases, underscored the notable prognostic predictive capacity of C1QBP (Figure 7a, Figures S9–S11). To elucidate the impact of C1QBP on osteoblastic cells, we partitioned these cells into two cohorts based on the median expression levels of C1QBP (Figure 7b), revealing the outcomes of differential gene analysis through a volcano plot (Figure 7c). Our GSVA analysis identified a series of upregulated metabolic and functionally related pathways in osteoblastic cells with elevated C1QBP expression, including FAM, intercellular communication, protein transport, gene expression regulation, and cell division and proliferation (Figure 7d). The GO analysis of differentially expressed genes indicated that C1QBP is primarily enriched in various biological processes, including cytoplasmic translation, extracellular matrix organization, and extracellular structure organization, implying a crucial role in protein synthesis and tissue organization. Concerning cellular components, C1QBP predominantly associates with the extracellular matrix and ribosomes, indicating a potential association with the secretion of extracellular vesicles and functionality within various cellular organelles. In the realm of molecular function, C1QBP predominantly correlates with structural constituents of ribosomes and the extracellular matrix, and cadherin binding (Figure 7e). The KEGG enrichment analysis revealed primary enrichment in cancer‐associated pathways, including ribosome, proteoglycans in cancer, and the PI3K‐Akt signaling pathway (Figure 7f). These results underscore C1QBP's pivotal role, especially regarding metabolic processes, cancer‐related pathways, and cellular signal transduction in osteoblastic cells, thereby illuminating its widespread potential impact on the pathogenesis and therapeutic strategies for this disease, providing valuable insights for subsequent research and treatment modalities.

### 3.9 C1QBP's role in remodeling TIME in OS

**FIGURE 8:**
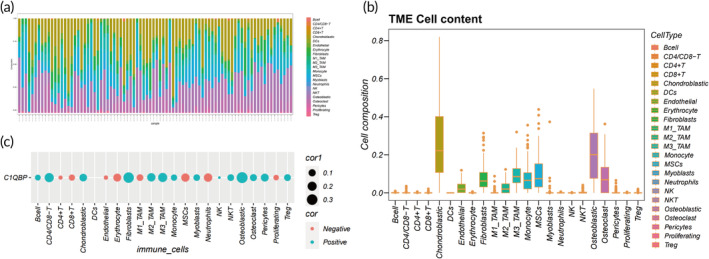
C1QBP's impact on the osteosarcoma tumor immune microenvironment. (a) Immune infiltration analysis for each sample. (b) Examination of cellular composition proportions across all samples. (c) Exploration of correlation between C1QBP and microenvironmental cells.

To elucidate the relationship between C1QBP and TIME in OS, we employed cibersortX to scrutinize the components of immune‐infiltrating cells within the TARGET‐OS data set, leveraging pre‐annotated single‐cell RNA data. Our analysis unveiled significant tumor heterogeneity within OS, as illustrated in Figure 8a. The predominant cellular constituents within the overarching TIME comprised osteoblasts, chondrocytes, monocytes/macrophages, fibroblasts, among others (Figure 8b). Importantly, correlations were observed between C1QBP and the majority of infiltrating cells within the OS TIME (Figure 8c), suggesting a potential role for C1QBP in modulating the TIME and influencing the progression of OS.

### 3.10 Implications of elevated C1QBP expression in multidrug resistance in OS

**FIGURE 9:**
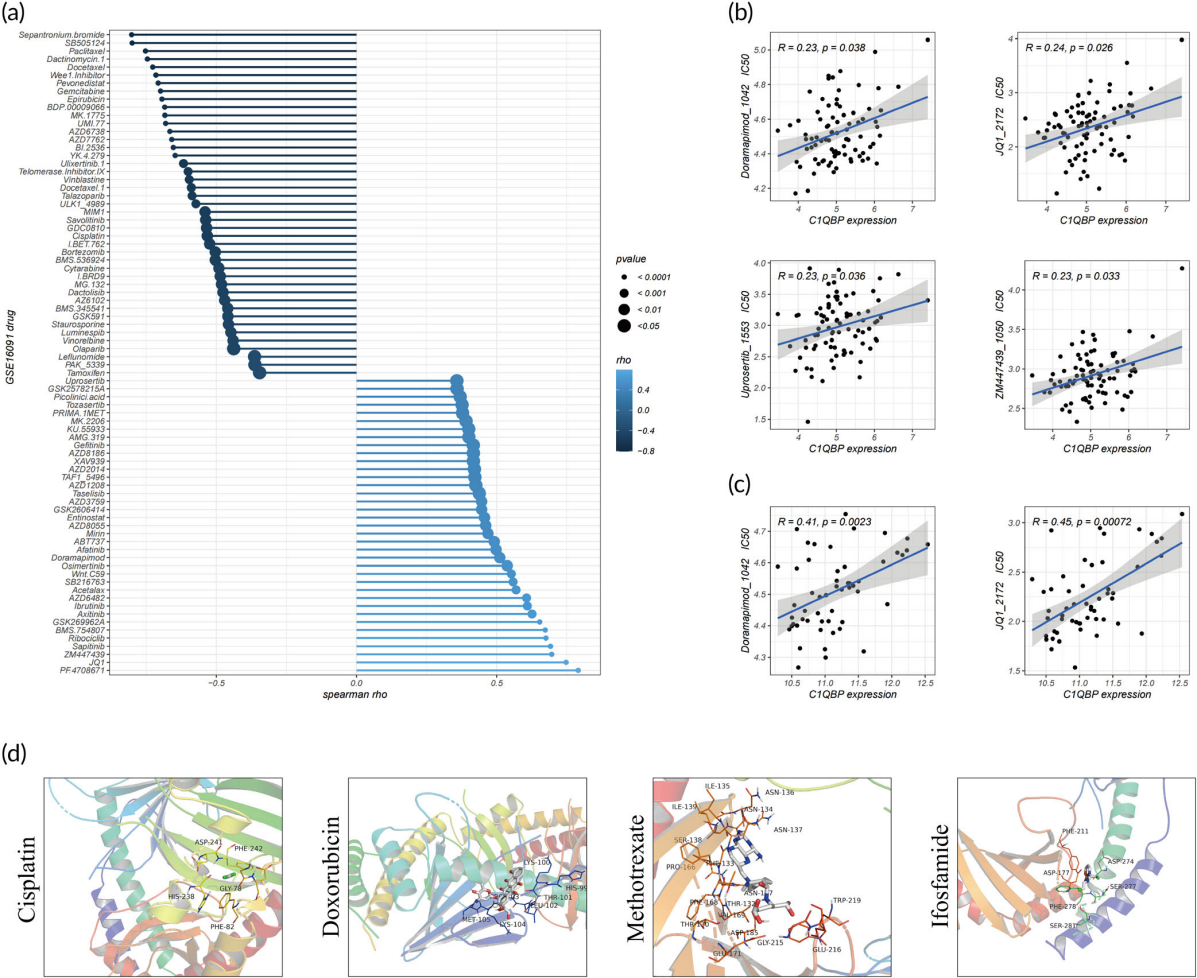
Elevated C1QBP levels correlate with increased drug resistance in osteosarcoma. (a–c) Correlation of C1QBP with IC_50_ values across various drugs in the GSE16091 (a), TARGET (b), and GSE21257 (c) databases. (d) Molecular docking analysis of C1QBP with primary osteosarcoma treatment drugs.

Drug resistance exerts a substantial impact on the prognostic outcomes of OS patients. Previous research has postulated a crucial role of FAM in drug resistance, with C1QBP potentially serving a distinctive role within this framework.^18^, ^52^ To explore the impact of C1QBP on drug sensitivity, we utilized the OncoPredict algorithm to transform gene expression data from the GSE16091, TARGET, and GSE21257 OS data sets into matrices reflective of drug sensitivity. Our analysis revealed a correlation between elevated C1QBP expression and augmented IC_50_ values for various drugs (Figure 9a–c), implying a potential involvement of C1QBP in engendering multidrug resistance in OS. Moreover, molecular docking studies disclosed interactions between C1QBP and first‐line OS drugs, uncovering significant affinities between C1QBP and cisplatin, doxorubicin, methotrexate, and ifosfamide (Figure 9d). These discoveries highlight C1QBP as a potential therapeutic target in OS management.

### 3.11 Potential facilitation of OS progression by C1QBP through the induction of monocyte differentiation into M2 and M3 macrophages

Upon integrating bulk and single‐cell data, alongside a review of existing literature, the intricate association between C1QBP and the mononuclear phagocyte system becomes evident. Subsequently, we isolated the mononuclear phagocyte system, reduced its dimensionality, executed clustering, and mitigated batch effects. Our examination of the impact of varying resolutions on cell clustering (Figure S12a) informed the selection of an optimal resolution. We categorized the mononuclear phagocyte system into four primary groups: M1 macrophages, M2 macrophages, M3 macrophages (FABP4+ macrophages), monocytes, and DCs (Figure S12b–d). M1 macrophages demonstrated elevated CD86 expression, M2 macrophages exhibited significantly heightened MRC1 (CD206) levels, M3 macrophages expressed robust FABP4 levels, and DCs showcased pronounced CD1C expression (Figure S12d). Consistent with bulk data, single‐cell data revealed elevated C1QBP expression in M2 and M3 macrophages, contrasted with its lower expression in M1 macrophages (Figure S12d). Heatmaps showcased divergent expression profiles of the mononuclear phagocyte system under varying states (Figure S12e).

**FIGURE 10:**
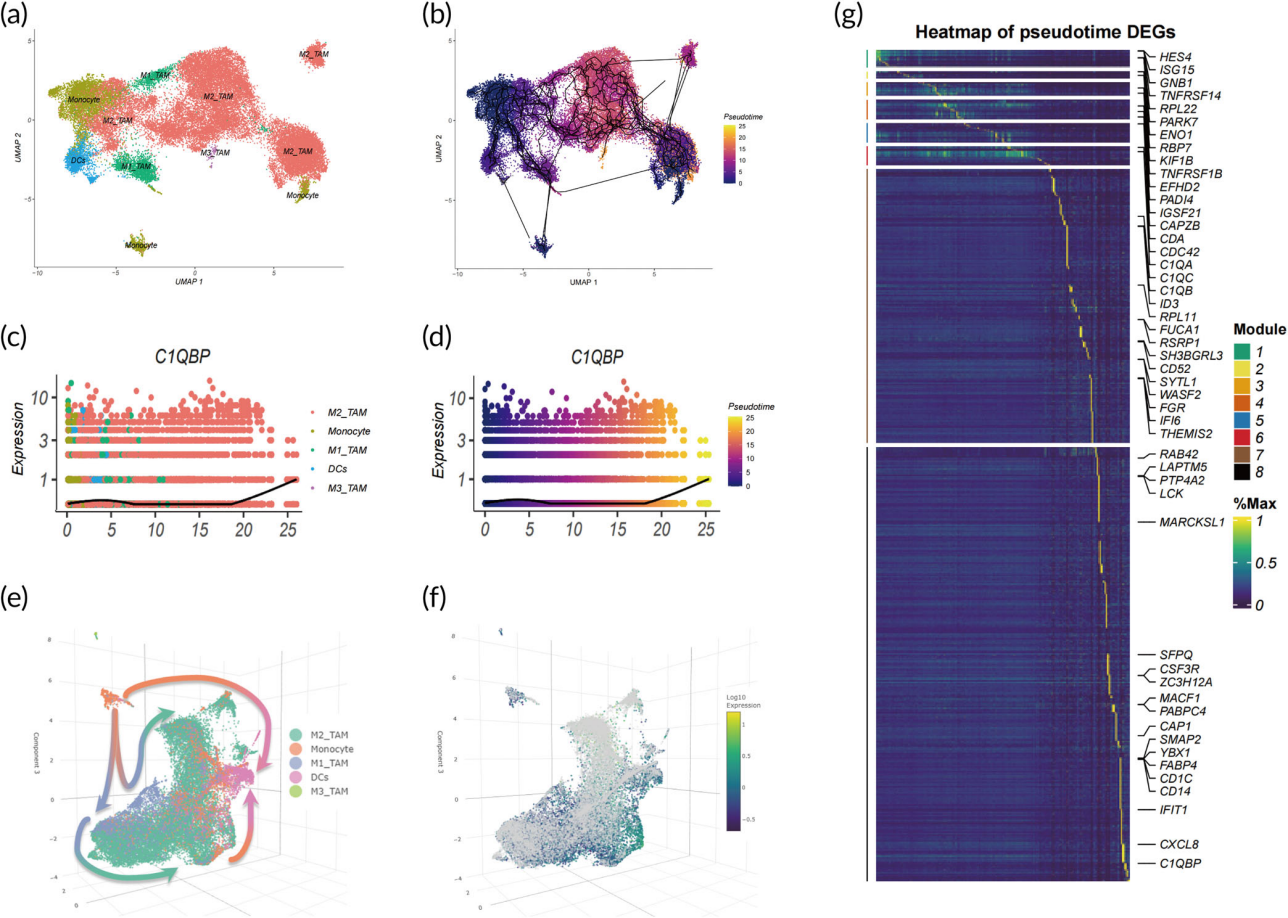
C1QBP's impact on macrophage polarization. (a–d) Monocle 3 trajectory plots depicting dynamic changes in mononuclear phagocyte system subpopulations (a), time course (b), and C1QBP expression levels (c and d). (e and f) 3D Monocle 3 trajectory plots of mononuclear phagocyte system subpopulations (e) and C1QBP expression levels (f). (g) Hierarchical clustering heatmaps displaying marker genes associated with developmental time and cell subpopulations generally (g).

Utilizing the Monocle 3 algorithm for the trajectory analysis of the mononuclear phagocyte system, we sought insights into macrophage polarization within OS. Employing Monocle 3, we organized cells along developmental trajectories, visualizing distinct cell types and their respective differentiation timelines within a two‐dimensional space (Figure 10a–f). Notably, C1QBP expressed prominently in M2 and M3 macrophages and DCs. The results from Monocle 3 provided a clearer depiction of the exhaustive differentiation process, extending from monocytes to M2 macrophages, M3 macrophages, and DCs (Figure 10b). Strikingly, a noticeable upregulation of C1QBP was observed during the transition from monocytes to M2 and M3 macrophages (Figure 10c–f). Through analyzing the gene expression heatmap during the differentiation process, C1QBP was found to be predominantly upregulated in the latter stages of differentiation (Figure 10g). Consequently, it is posited that C1QBP facilitates OS progression by steering the polarization of monocytes/macrophages toward the M2 and M3 phenotypes.

### 3.12 Analyzing the influence of C1QBP on proliferation, apoptosis, and drug resistance in OS cells via in vitro studies

**FIGURE 11:**
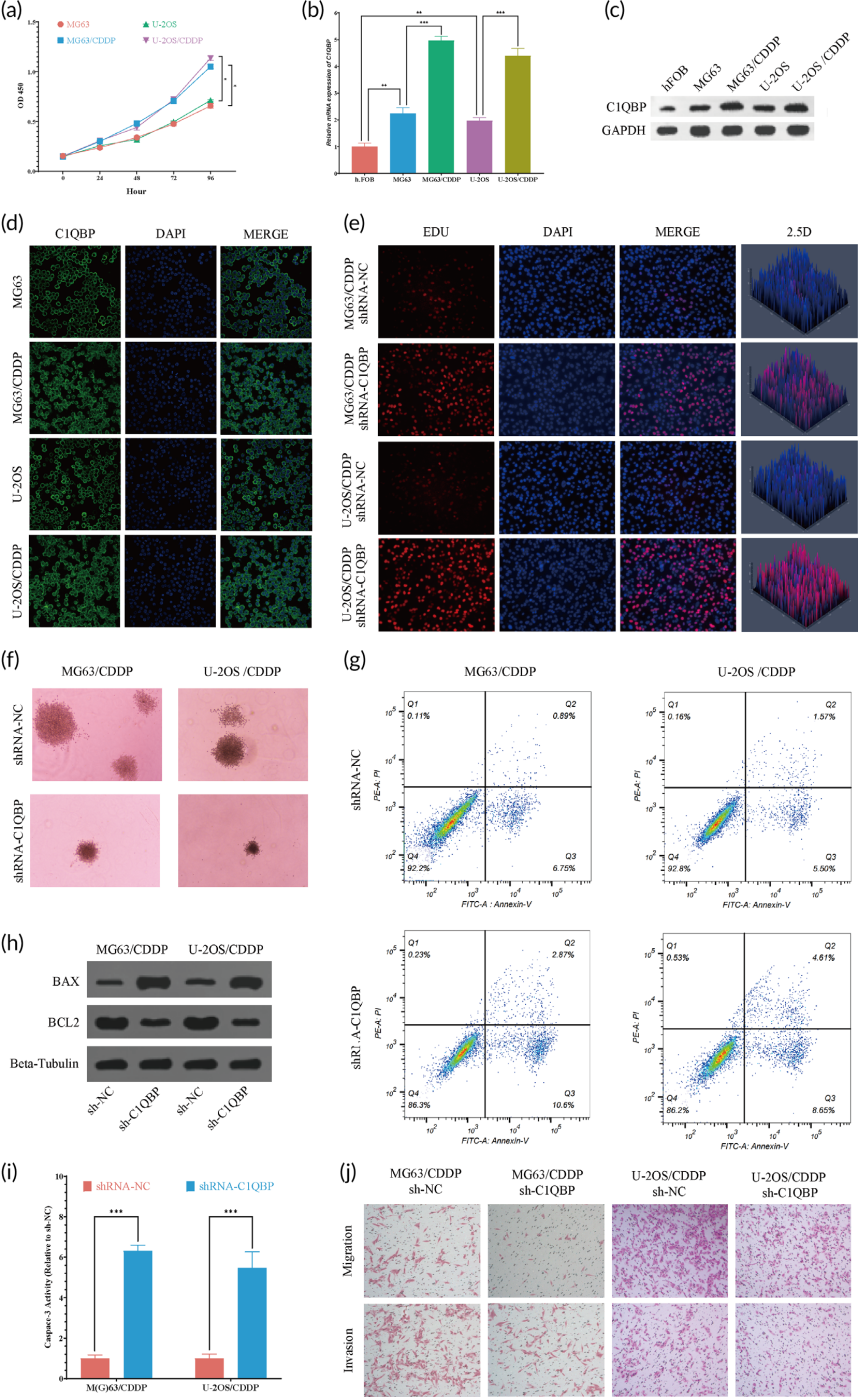
Experimental confirmation of C1QBP's pro‐oncogenic effect in vitro. (a) Growth assessment in osteosarcoma‐sensitive and drug‐resistant cell lines using CCK‐8. (b) C1QBP expression evaluation in osteosarcoma and cisplatin‐resistant osteosarcoma cells via qRT‐PCR. (c) Analysis of protein expression levels by Western blot. (d) Confocal microscopy examination of C1QBP expression in both sensitive and drug‐resistant osteosarcoma cells. (e and f) Observations of EdU staining assay (e) and 3D culture illustrating (f) the impact of C1QBP knockdown on drug‐resistant osteosarcoma cells. (g) Apoptosis assessment in osteosarcoma cells after 48 h using flow cytometry. (h) Western blotting to assess apoptotic protein levels. (i) An assay for enzymatic activity of caspase‐3 was conducted. (j) Transwell migration and invasion assays. *p < 0.05, **p < 0.01, ***p < 0.001. qRT‐PCR, quantitative reverse transcription‐polymerase chain reaction.

Cisplatin‐resistant OS cell lines, MG63/CDDP, and U‐2OS/CDDP were successfully generated using a gradual escalation methodology, as detailed in Figure S13a. To assess the cisplatin sensitivity of these established cell lines, the CCK‐8 assay was employed. The MG63/CDDP and U2‐OS/CDDP lines exhibited half‐maximal inhibitory concentration (IC_50_) values for cisplatin of 49.13 μM and 73.92 μM, respectively, compared to their parental cells, MG63 and U‐2OS, which displayed IC_50_ values of 7.37 μM and 12.22 μM, respectively, underscoring the effective establishment of cisplatin‐resistant OS cell lines (Figure S13b). Additionally, the drug‐resistant OS cells demonstrated markedly accelerated growth rates at 72 and 96 h, compared to the parental cells (Figure 11a).

Quantitative reverse transcription‐polymerase chain reaction (qRT‐PCR) and Western blot analyses indicated a significant increase in C1QBP expression levels in MG63 and U‐2OS OS cells compared to the normal osteoblast cell line hFOB. Additionally, relative to their parental cells, MG63/CDDP and U‐2OS/CDDP cells displayed a substantial upregulation in C1QBP expression, with especially elevated levels noted in U‐2OS/CDDP cells (Figure 11b,c and Figure S13c). Immunofluorescence revealed C1QBP localization at the membrane (Figure 11d).

To further investigate the impact of C1QBP on OS, we formulated a specific shRNA targeting C1QBP (shRNA‐C1QBP). Results unequivocally indicated that shRNA‐C1QBP notably inhibited C1QBP expression in OS cells (Figure S13d). Moreover, following cisplatin treatment at various concentrations across different OS cell groups, the shRNA‐C1QBP group exhibited a notable decrease in cell viability and IC_50_ values compared to the shRNA‐NC group (Figure S13e,f).

Upon shRNA‐C1QBP administration, a significant reduction in the proliferation capability of cisplatin‐resistant OS cells was observed following a 48‐h shRNA‐C1QBP treatment within a 3D culture environment and EdU detection (Figure 11e,f).

Notably, inducing tumor cell apoptosis is crucial in cancer therapy and is a primary target in addressing drug resistance. Figure 11f,g depicts that cisplatin‐resistant OS cells treated with shRNA‐NC for 48 h exhibited apoptosis rates of 7.07 ± 0.7% and 7.64 ± 0.8%, respectively. In contrast, a 48‐h treatment with shRNA‐C1QBP led to a marked increase in apoptosis rates, reaching 13.26 ± 1.5% and 13.47 ± 1.3% (Figure 11g and Figure S13g). The results of Western blot assays indicate that the knockout of the *C1QBP* gene in two cell lines led to an increase in the expression of BAX protein and a decrease in the expression of BCL2 protein (Figure 11h). Furthermore, the enhancement in Caspase‐3 activity, as confirmed by Caspase‐3 activity assays, substantiates the effect of *C1QBP* gene knockout (Figure 11i). These findings collectively support the regulatory role of the *C1QBP* gene in the apoptosis process in OS cell lines.

The results from the Transwell assays revealed that the migration and invasion capacities of MG63/CDDP and U‐2OS/CDDP cell lines were significantly reduced following the knockout of the *C1QBP* gene. This finding suggests that the *C1QBP* gene may play a crucial role in regulating the migration and invasion of these OS cell lines (Figure 11j).

### 3.13 In vivo evaluation of the effect of C1QBP on drug resistance, apoptosis, and proliferation in OS cells

**FIGURE 12:**
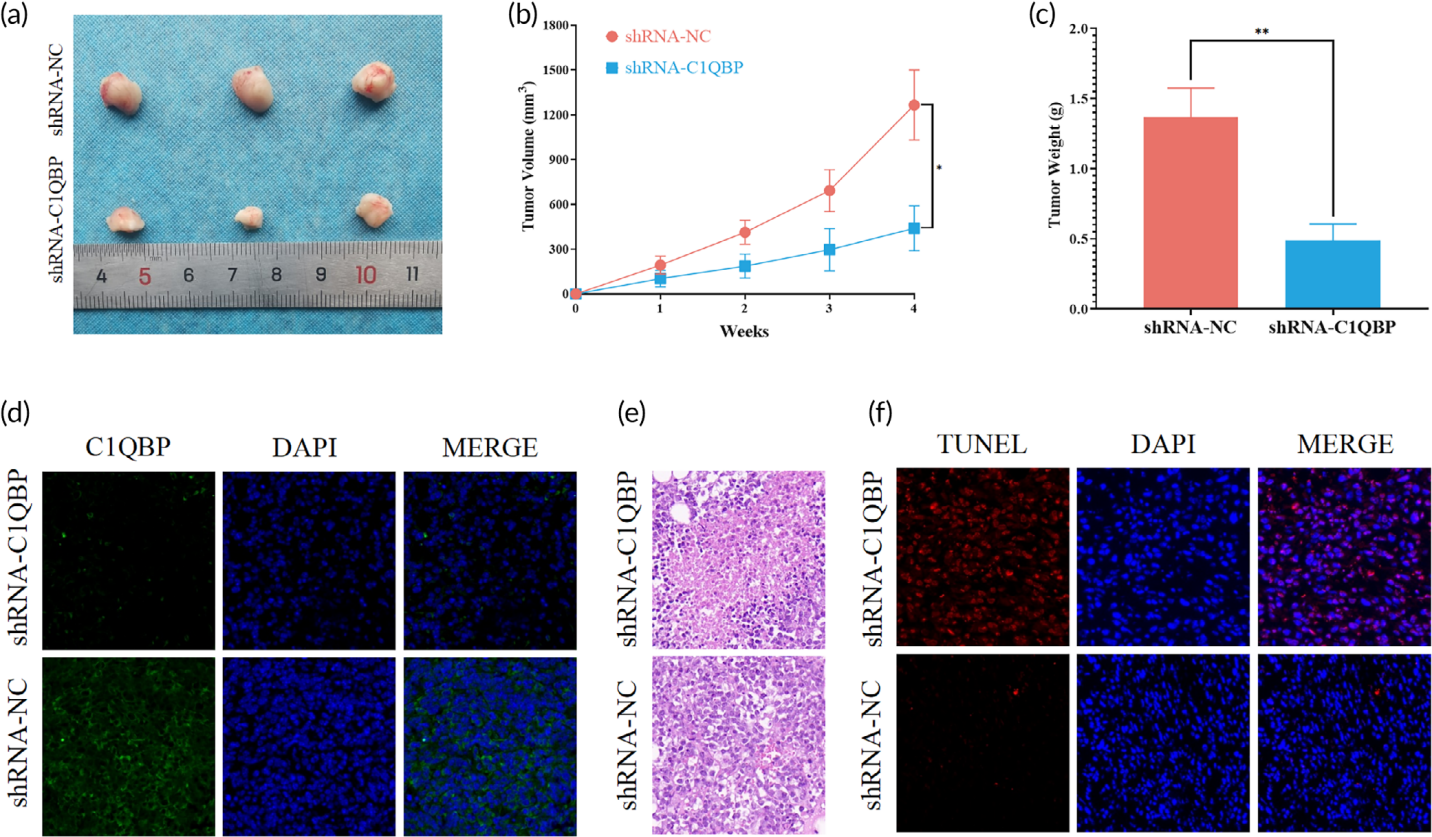
Experimental confirmation of C1QBP's pro‐oncogenic effect in vivo. (a) Images of subcutaneous tumors in nude mice subjected to various treatments. (b) Growth curves of tumor volume for each group, calculated using *V* = (*a* × *b* ^2^)/2. (c) Comparison of tumor weights among different mouse groups. (d) Assessment of C1QBP expression in excised tumor tissues via immunofluorescence. (e) Hematoxylin and eosin (HE) staining for histological analysis of excised tumor tissues. (f) Detection of TUNEL expression in excised tumor tissues through immunofluorescence.

Upon establishing the integral role of C1QBP in various aspects of OS phenotypes, including drug resistance, apoptosis, and proliferation through preceding in vitro experiments, our inquiry was extended to elucidate the influence of C1QBP on OS via the development of a subcutaneous xenograft model in nude mice. The experimental results indicated a significant decrease in both tumor mass and volume in the shRNA‐C1QBP group relative to the shRNA‐NC group (Figure 12a–c).

Analysis of tumor tissue sections from the internal environment revealed a pronounced downregulation of C1QBP expression in the shRNA‐C1QBP group, as demonstrated in Figure 12d and Figure S13h. Moreover, H&E staining (Figure 12e) revealed evident damage and a lax cellular architecture in tumor cells of the shRNA‐C1QBP group. To validate the proapoptotic effect of C1QBP in OS cells in vivo, TUNEL staining was employed to confirm cellular apoptosis. As indicated in Figure 12f and Figure S13i, nuclei were stained blue, while apoptotic cells were identified by red staining. Remarkably, an appreciable increase in apoptosis was observed in the shRNA‐C1QBP‐treated cells relative to the shRNA‐NC group. These results cohesively affirm the concordance between TUNEL staining in tumor tissue sections from the internal milieu and the induction of cellular apoptosis noted in shRNA‐C1QBP‐treated cells in vitro.

### 3.14 Exosome‐mediated differentiation of the mononuclear phagocyte system induced by drug‐resistant OS cell lines

**FIGURE 13:**
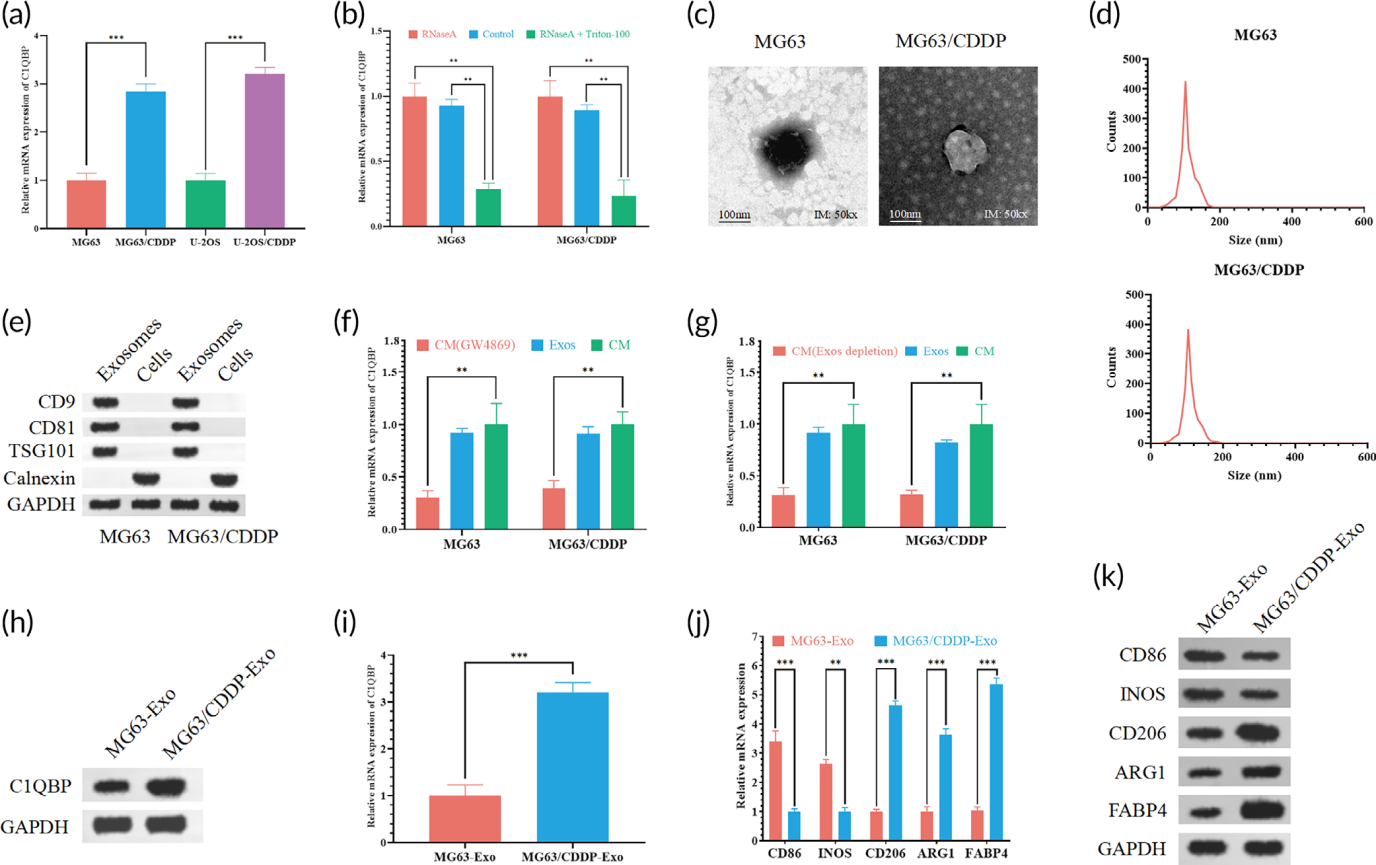
Promotion of macrophage polarization by exosomes from drug‐resistant osteosarcoma cell lines. (a) qPCR analysis indicating C1QBP expression levels in conditioned medium (CM) derived from parental and drug‐resistant osteosarcoma cell lines. (b) qPCR data demonstrating C1QBP expression in MG63 and MG63/CDDP cell lines under various treatments: control medium, RNase A (2 mg/mL), and a mixture of RNase A and Triton X‐100 (0.1%) for 0.5 h. Phenotypic characteristics of exosomes from HCT‐8, HT29, LoVo, and Caco‐2 cells are examined via transmission electron microscopy (c) and NanoSight nanoparticle tracking analysis (d). (e) Protein blot analysis identifying typical exosome markers (CD9, CD81, and TSG101) in exosomes from osteosarcoma cell lines. Evaluation of C1QBP expression in CM from osteosarcoma cells post‐exosome depletion using GW4869 treatment (an exosome secretion inhibitor) (f) and ultracentrifugation (g), with CM from MG63 and MG63/CDDP cells as references. Differential C1QBP expression in exosomes from osteosarcoma cells and cisplatin‐resistant osteosarcoma cells is portrayed through Western blot (h) and qRT‐PCR (i). (j and k) Employ qRT‐PCR and WB for evaluating the expression of M1, M2, and M3 macrophage marker genes. Significance: **p < 0.01, ***p < 0.001. qPCR, quantitative polymerase chain reaction; qRT‐PCR, quantitative reverse transcription‐polymerase chain reaction; WB, Western blot.

In an endeavor to discern the role of C1QBP in OS drug resistance and recurrence, C1QBP expression was assessed in the conditioned media (CM) derived from both sensitive and drug‐resistant OS cells. The CM from drug‐resistant OS cells manifested a comparatively elevated C1QBP expression relative to that from sensitive cell lines (Figure 13a). Notably, post RNase A treatment, C1QBP levels within the OS cell CM remained constant, while a significant diminution was observed upon combining RNase A with Triton X‐100 treatment (Figure 13b), suggesting that extracellular C1QBP is membrane‐encapsulated and not directly secreted.

Exosomes, extracted from the culture supernatants of MG63 and MG63/CDDP cells, were identified utilizing a synergistic approach involving transmission electron microscopy, protein immunoblot analysis, and Nanosight analysis. The extracted exosomes predominantly exhibited a circular or elliptical, membrane‐bound vesicular shape, characteristic of exosomes (Figure 13c). Nanoparticle tracking analysis displayed exosome diameters ranging between 30 and 200 nm, with a notable peak at 100 nm (Figure 13d).

Investigations into the presence of specific exosome surface protein markers, namely CD9, CD81, TSG101, and the negative control protein calnexin (associated with the endoplasmic reticulum), were conducted through Western blot experiments. The results evinced a substantial increase in the levels of the exosome surface protein markers CD9, CD81, and TSG101, while calnexin was undetected on the exosome surface (Figure 13e), thereby validating the successful isolation of exosomes from OS cells. The source of C1QBP was verified utilizing GW4869 to inhibit exosome secretion; the expression level of C1QBP in exosomes from OS cells was markedly higher than that from GW4869‐treated cells (Figure 13f).

Moreover, we employed qPCR assays to gauge the expression of C1QBP in total CM, exosome‐depleted CM (via high‐speed centrifugation), and the exosomes. A notably heightened C1QBP expression in both total CM and exosomes as opposed to exosome‐depleted CM (Figure 13g) substantiated the packaging of C1QBP into exosomes from OS cells. Further analysis of the differential expression of C1QBP in exosomes from OS cells and cisplatin‐resistant OS cells was executed. Both qRT‐PCR and Western blot results affirmed that C1QBP expression in exosomes from cisplatin‐resistant OS cells, MG63/CDDP, was significantly elevated relative to MG63 exosomes (Figure 13h,i). Furthermore, MG63/CDDP‐Exo treatment yielded an upregulation in CD206, ARG1, and FABP4 expression and a downregulation in CD86 and INOS expression in RAW264.7, signifying the promotion of macrophages polarization toward M2 and M3 phenotypes (Figure 13j,k).

## 4 DISCUSSION

OS, characterized by remarkable invasiveness, demonstrates a notably poor prognosis for patients experiencing recurrence or metastasis due to its pronounced heterogeneity^27^, ^53^, ^54^; furthermore, the event‐free survival rate for metastatic patients languishes below 20%.^3^, ^4^ A tangible link exists between the progression and grim prognosis of OS and chemotherapy resistance.^7^, ^8^ Cisplatin, a chemotherapy agent widely utilized, significantly impacts the treatment of various cancers, including OS^8^, ^55^, ^56^; however, the resistance mechanism of OS to this agent remains elusive. TIME involves a complex network and perpetual interactions between tumor cells—OS included—and it markedly affects tumor initiation, progression, metastasis, and drug resistance.^57^, ^58^, ^59^ Contemporary research accentuates the pivotal role of TIME in cancer pathogenesis, achieved via an intricate intercellular communication system encompassing both direct cell–cell interactions and classical paracrine signaling, mediated by cytokines and growth factors.^60^, ^61^, ^62^ Several studies have underscored that tumor cells can remodel their immune microenvironment, thereby inducing drug resistance.^59^, ^63^, ^64^ TIME has drawn substantial research and clinical focus, positioning itself as a compelling therapeutic target in oncology.^58^, ^65^ Thus, exploring OS progression and drug resistance mechanisms through the lens of TIME emerges as a promising research trajectory. In this study, we meticulously integrated multiple data sets from scRNA‐seq and bulk‐seq, exploring the heterogeneity of osteoblastic cells by contrasting differences between control bone and OS. Concurrently, through bioinformatic analysis of osteoblastic cells and TIME components, we elucidated the complexities within the tumor, presenting potential therapeutic targets for prospective OS treatments.

FAM, encompassing synthesis, breakdown, regulation, and utilization of fatty acids, plays pivotal roles in maintaining energy balance, constructing cell membranes, synthesizing hormones, mediating inflammatory responses, and storing lipids.^66^ The regulation of FAM satisfies energy requirements and influences various cancer cell aspects, including proliferation, growth, transformation, recurrence, and drug resistance.^67^, ^68^ Numerous recent reports underscore targeting FAM as a viable approach to counteract treatment resistance.^69^, ^70^, ^71^ Through integrating scRNA‐seq with Bulk‐seq databases, we identified a gene highly expressed in OS related to FAM, C1QBP, which is associated with survival prediction. C1QBP, a member of the FAM gene family, can regulate mitochondrial activity, affecting both tumors and immune cells, which has significant implications for cancer treatment.^72^, ^73^, ^74^ Utilizing various analytical approaches, including GO, KEGG, GSVA, and GSEA, we explored the potential functions of C1QBP in OS, which involve controlling the extracellular matrix, composing extracellular structures, synthesizing proteins, and more, thereby highlighting its crucial role within the OS immune microenvironment. Additionally, our investigation into chemotherapy drug sensitivity and binding affinity revealed that C1QBP not only elevates the IC_50_ values for various drugs but also demonstrates significant affinity toward frontline chemotherapy agents. Noteworthily, our in vitro and in vivo experiments validate these bioinformatics discoveries, with C1QBP observed to possess attributes that confer resistance to apoptosis, enhance tumor migration and invasiveness, and play a critical role in developing cisplatin resistance in OS.

Through a meticulous analysis of comprehensive cell‐to‐cell communication patterns, we unveiled the intricate network of interactions within the OS immune microenvironment. Malignant osteoblasts engage in multifaceted interactions with non‐tumor cells via diverse mechanisms. To discern which intercellular communication patterns might contribute to OS progression, we conducted an exhaustive comparative analysis of the communication profiles between control and OS cells. Significantly, signaling pathways, including IFN‐II, BMP, APELIN, ncWNT, OSTN, NRG, and others, are notably upregulated in OS tissues. Prior studies have highlighted a significant association between these elevated pathways and processes of FAM, tumor progression, drug resistance, and macrophage polarization, pointing toward the crucial involvement of FAM and macrophages in OS progression and drug resistance.^43^, ^44^, ^45^, ^46^, ^47^, ^48^, ^49^, ^50^, ^51^ Macrophages, pivotal players in cancer progression and metastasis, exhibit diverse functions: M1 macrophages demonstrate tumor cell phagocytic activity,^75^, ^76^ while M2 macrophages, including tumor‐associated macrophages, promote tumor growth, invasion, therapeutic resistance, and immune evasion.^77^, ^78^, ^79^ M2 macrophages within the tumor immune microenvironment can mediate the chemotherapy resistance of tumor cells, thus impacting patient prognosis.^77^ In lung metastatic OS lesions, infiltration of proinflammatory M3 macrophages (FABP4+) is observed, and these M3 macrophages seemingly contribute to establishing the proinflammatory TIME within the lung metastatic OS cells.^27^ Our comprehensive analysis of scRNA‐seq and bulk‐seq data revealed elevated C1QBP expression levels in M2 and M3 macrophages and reduced expression in M1 macrophages. Furthermore, pseudotime analysis findings suggest that C1QBP promotes the polarization of monocytes/macrophages toward the M2 and M3 phenotypes.

Exosomes, diminutive, nanometer‐scale, membrane‐bound particles, facilitate intercellular communication through the conveyance of bioactive molecules between adjacent cells, emerging as pivotal mechanisms for cellular information exchange.^61^, ^80^, ^81^ Remarkably, exosomes emanating from OS cells influence numerous aspects of tumor progression, encompassing proliferation, recurrence, metastasis, and the emergence of drug resistance.^81^, ^82^, ^83^ In vitro studies reveal that exosomes, discharged from cisplatin‐resistant OS cells, bear a significant load of C1QBP, indicating that these extracellular vesicles might directly impact monocytes/macrophages, thus influencing macrophage polarization. Subsequent experiments validated this hypothesis, demonstrating that C1QBP‐rich exosomes can instigate the polarization of macrophages into M2 and M3 phenotypes, spotlighting the critical role of drug‐resistant OS cells in modulating macrophage polarization via C1QBP.

This research cogently delineates C1QBP's formidable impact in augmenting both the proliferation and anti‐apoptotic traits of OS cells and significantly fortifying their resistance to cisplatin. Notably, cisplatin‐resistant OS cells can secrete C1QBP through exosomes, thereby instigating a pronounced polarization of macrophages into M2 and M3 phenotypes. Consequently, C1QBP emerges as a paramount factor influencing cancer drug resistance and potentially regulating macrophage polarization, representing dual elements in OS progression. Given these findings, as subsequent research endeavors to unravel the mechanisms through which C1QBP propels OS, exhaustive interventions targeting C1QBP might unveil potential strategies to enhance cisplatin sensitivity in OS cells, thereby presenting innovative therapeutic pathways and strategies to ameliorate the prognosis for OS patients.

## 5 CONCLUSION

This study meticulously delineates the OS tumor immune microenvironment and probes the mechanisms intrinsic to the progression and drug resistance of OS. The analysis unveils that *C1QBP*, a gene significantly expressed and prognostically pertinent within the context of FAM, holds a crucial role in OS. Through both in vitro and in vivo experiments, C1QBP is substantiated to exert a profound impact on cell proliferation, anti‐apoptotic responses, migration, invasion, tumor immune microenvironment modulation, cisplatin resistance promotion, and potentially on macrophage polarization. Therefore, C1QBP is spotlighted as a promising therapeutic target, paving the way for potential interventions that could notably amplify treatment outcomes for OS patients.

## AUTHOR CONTRIBUTIONS

**Xin Wu:** Data curation (equal); formal analysis (equal); validation (equal); visualization (equal). **Ning Tang:** Investigation (equal); methodology (equal); resources (equal); software (equal); writing – original draft (equal). **Qiangqiang Zhao:** Conceptualization (equal); supervision (equal); writing – review and editing (equal). **Jianbin Xiong:** Funding acquisition (equal); project administration (equal).

## FUNDING INFORMATION

This work was supported by the National Natural Science Foundation of China (no. 82260365, 82372315), Liuzhou Science and Technology Program (2021YB0104B060) and Hunan Provincial Innovation Foundation for Postgraduate (CX20230296).

## CONFLICT OF INTEREST STATEMENT

The authors declare no conflicts of interest.

## Supporting information


**Data S1.** Supporting information.

## Data Availability

Single‐cell data were retrieved from the GEO database (GSE152048, GSE162454, GSE169396, and GSE217792), and bulk data were obtained from GEO (GSE16091 and GSE21257) and the TARGET database. Other data that support the findings of this study are available from the corresponding author upon reasonable request.
