# Supplementary material for: Spatiotemporal evolutionary process of osteosarcoma immune microenvironment remodeling and C1QBP‐driven drug resistance deciphered through single‐cell multi‐dimensional analysis
Source: Bioeng Transl Med. 2024 Apr 10;9(5):e10654. doi: 10.1002/btm2.10654 (PMC11561849; doi:10.1002/btm2.10654)
Supplement: Supplementary file 1 — Data S1. Supporting information. [file BTM2-9-e10654-s001.docx]

**Supplementary Figures**


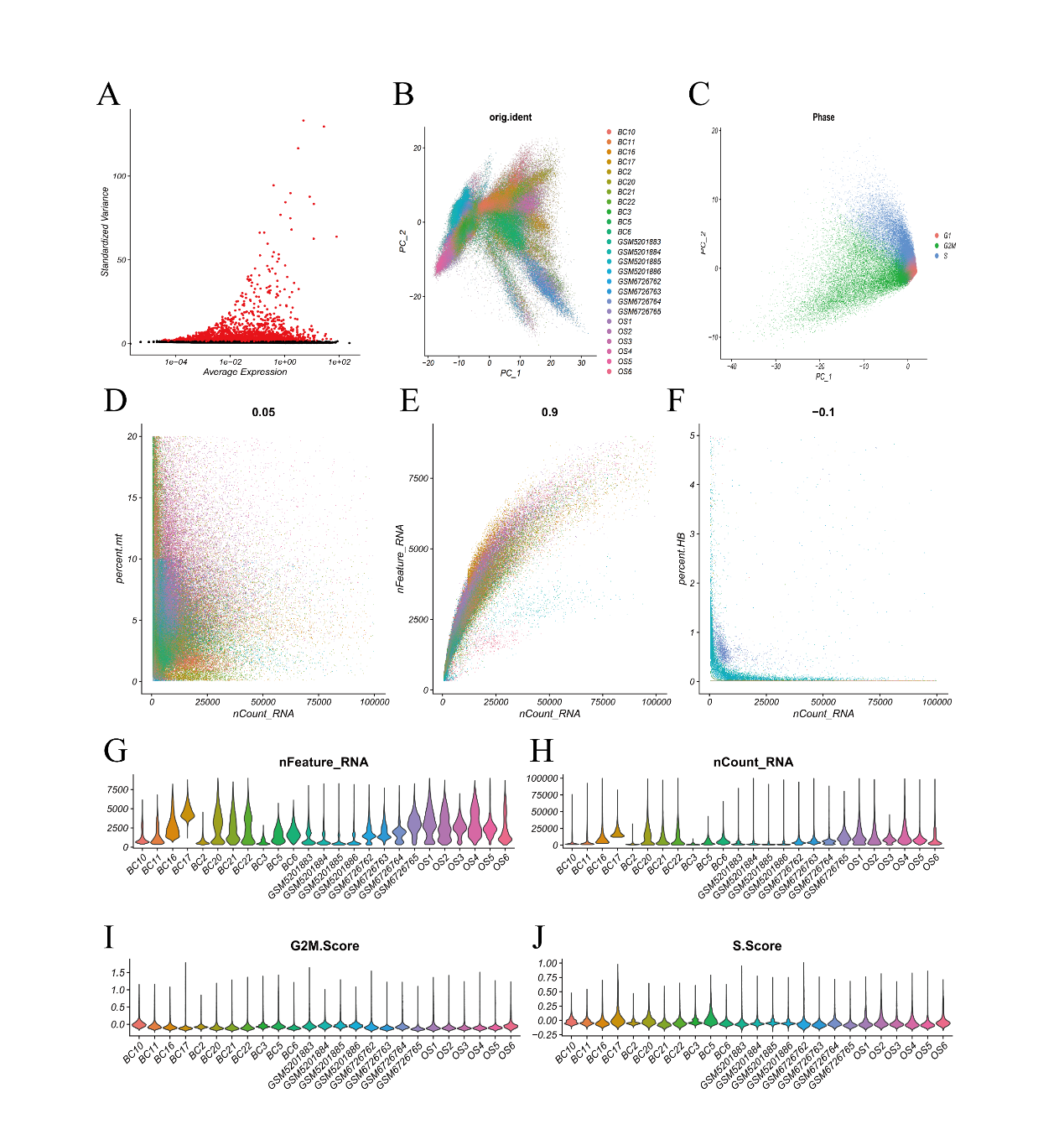


**Figure S1. Quality Control Analyses.** (A) Identification of highly variable genes across cells. (B) PCA plot, color-coded for 25 samples. (C) PCA plot representing cell cycle status across the 25 samples. (D) Scatter plot of the relationship between the total sum of gene expression levels per cell and the percentage of mitochondrial genes. (E) Scatter plot of the correlation between the total gene expression sum per cell and the percentage of gene counts. (F) Scatter plot of the relationship between the total gene expression sum per cell and the percentage of red blood cell gene counts. (G) Gene counts per sample. (H) Total gene expression levels per sample. (I) G2M score levels per sample. (J) S score levels per sample.


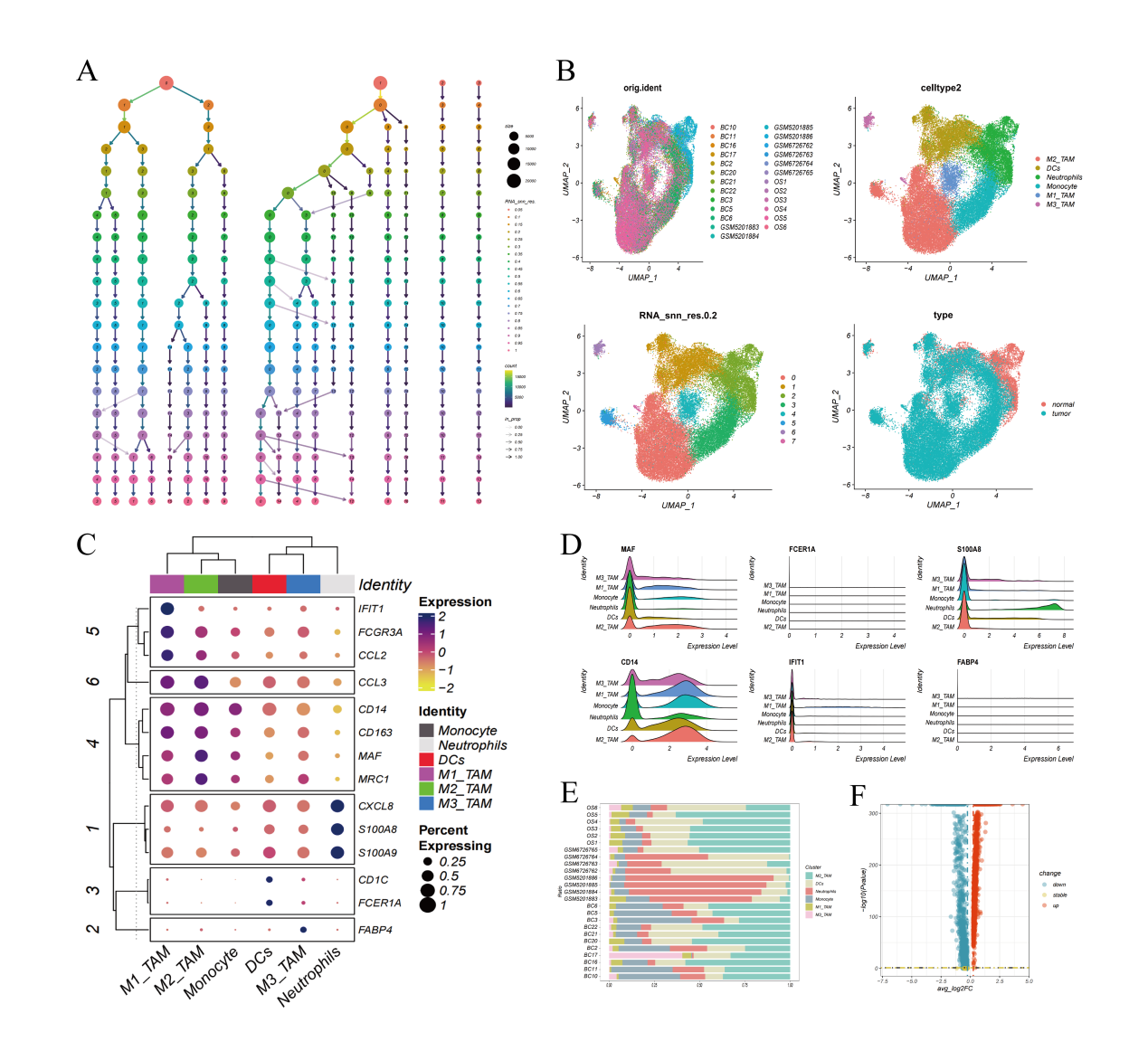


**Figure S2. Myeloid Cells in the Osteosarcoma Immune Microenvironment.** (A) Subclustering of all cells at various resolutions. (B) UMAP plots illustrating distinctions among samples, cell types, clusters, and clinical categories. (C) A bubble diagram displaying expression patterns of classic myeloid cell-related marker genes. (D) Ridge plots showing the distribution of classic myeloid cell-related marker gene expressions. (E) Relative proportions of each myeloid cell subtype across the 25 samples. (F) A volcano plot highlighting differential gene expression between control-TILs and OS-TILs, with red genes indicating upregulation in OS samples.


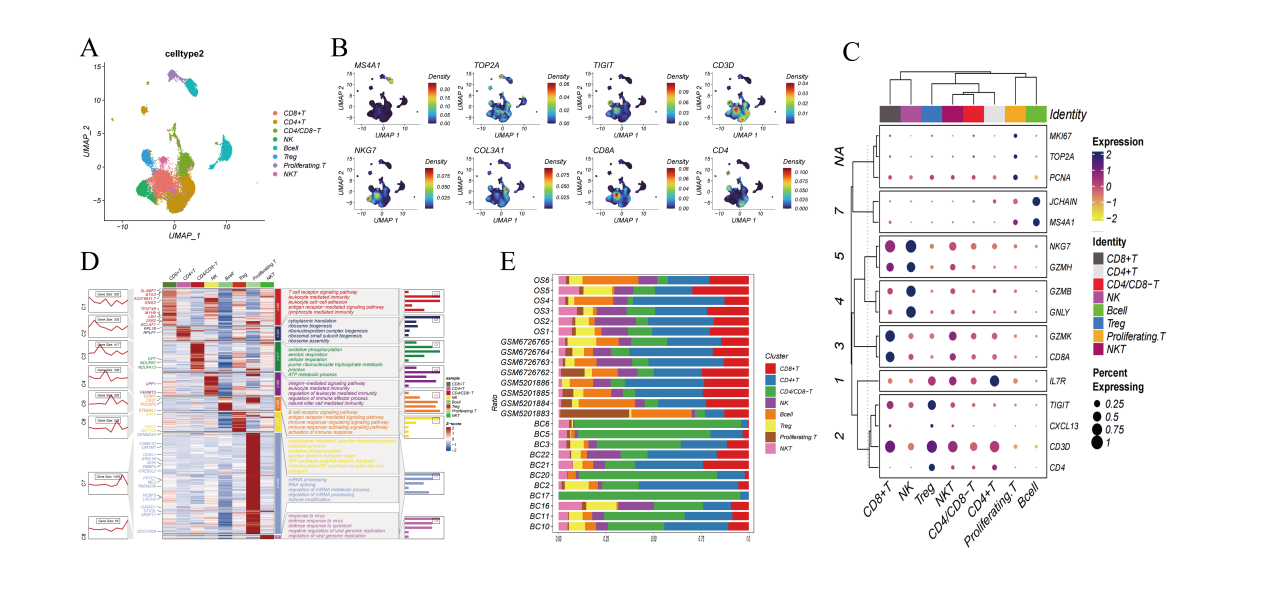


**Figure S3. Comprehensive Analysis of TILs within the Osteosarcoma Immune Microenvironment.** (A) UMAP plots visualizing distinct cell types. (B) A density plot presenting expressions of classic TIL-related marker genes. (C) Bubble plot illustrating the expression patterns of classic TIL-related marker genes. (D) Heatmap depicting DEGs and conducting GO analysis among the eight TIL subclusters. (E) Proportions of each TIL subtype across the 25 samples.


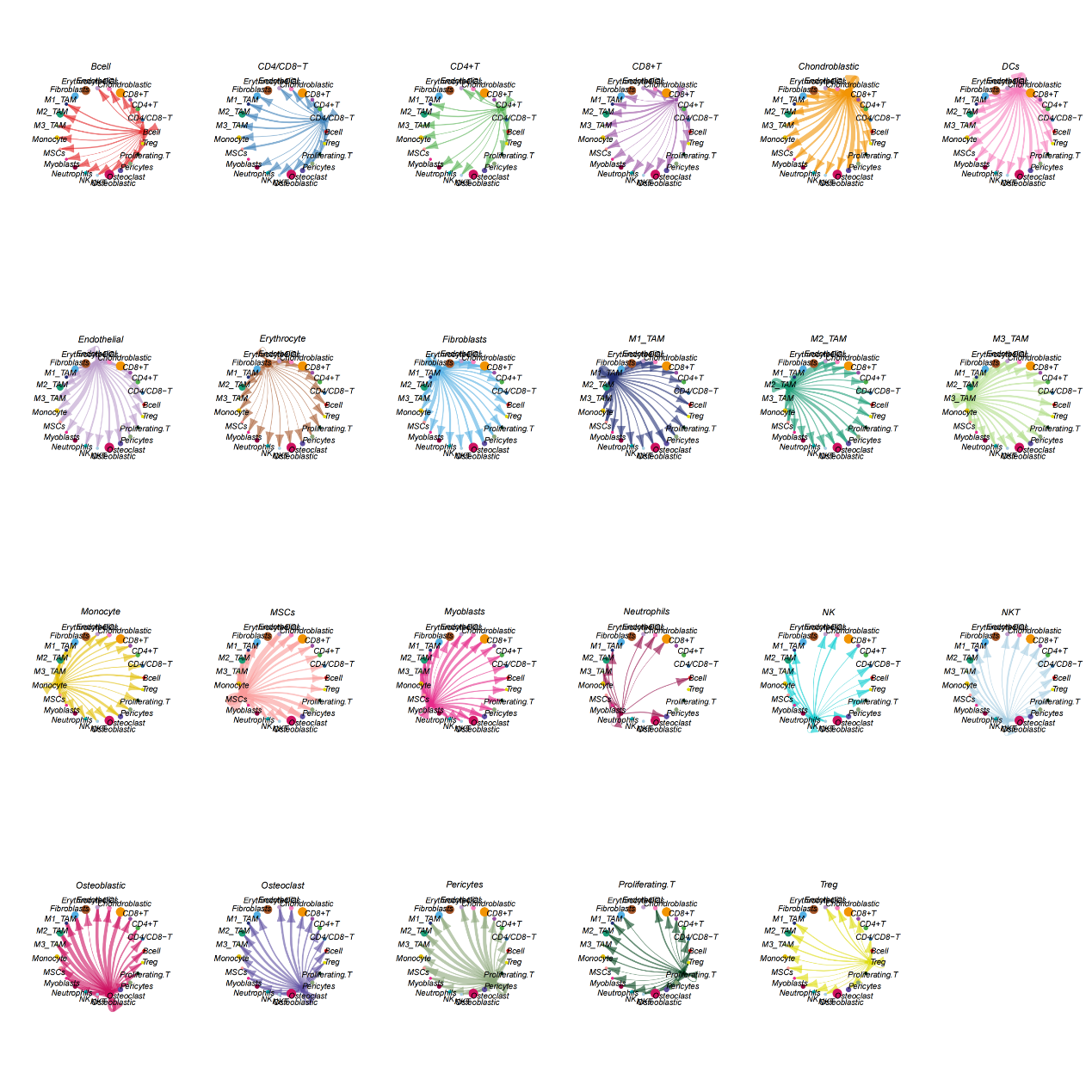


**Figure S4. Visualizing Signaling Quantity from Each Cell Subpopulation.**


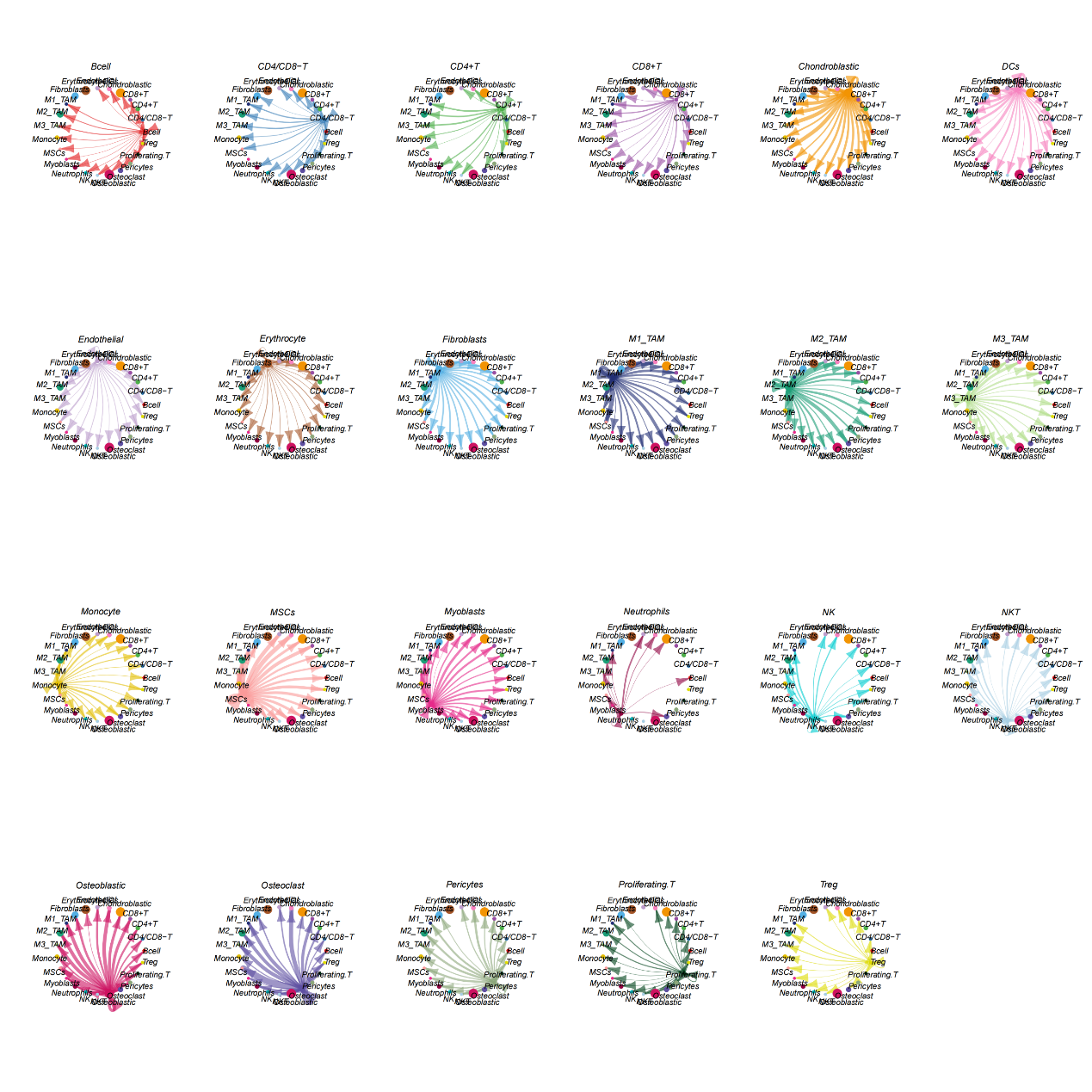


**Figure S5. Visualizing Signaling Intensity from Each Cell Subpopulation.**


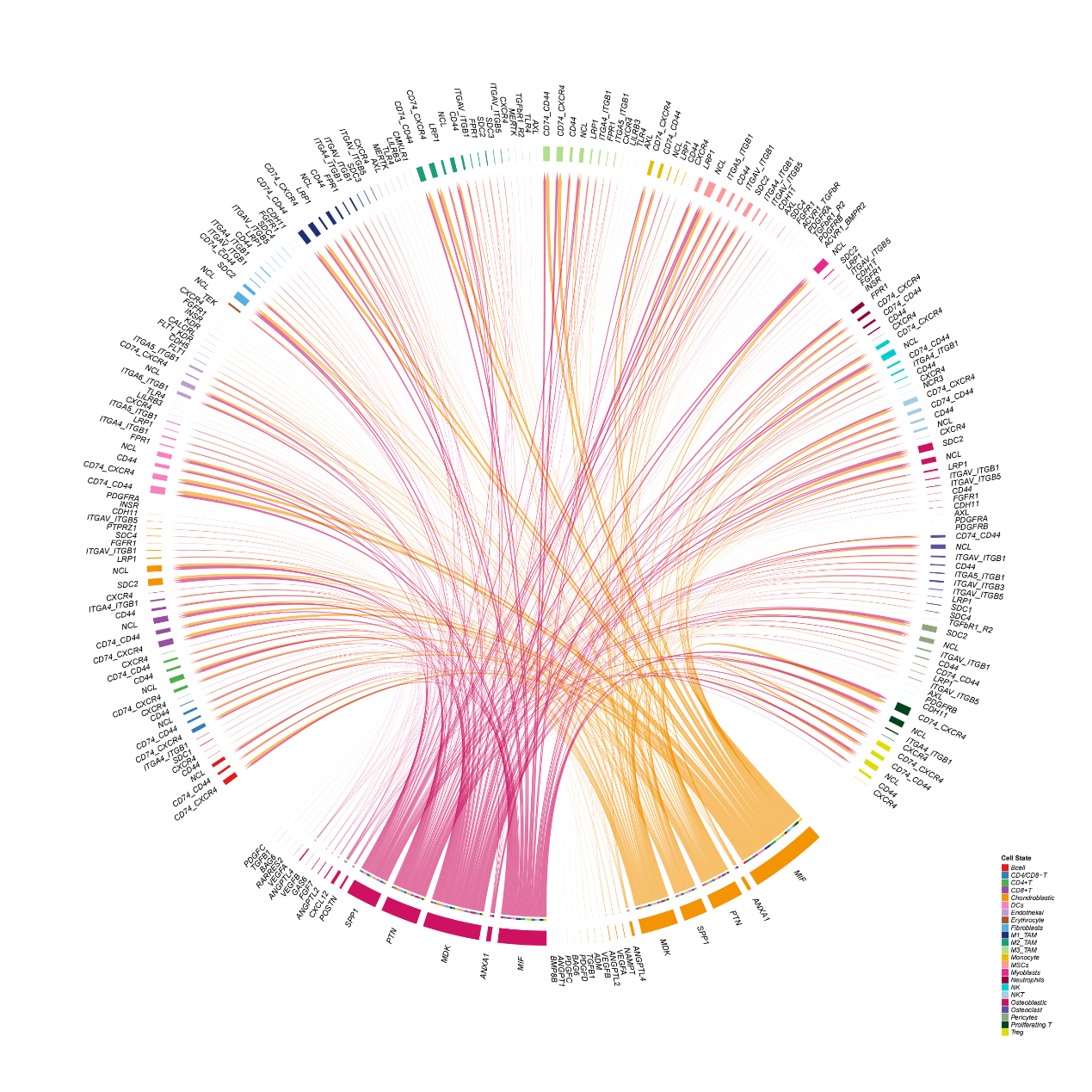


**Figure S6. Influence of Osteoblast and Chondroblast Cells as Signaling Senders on Other Cell Subpopulations.**


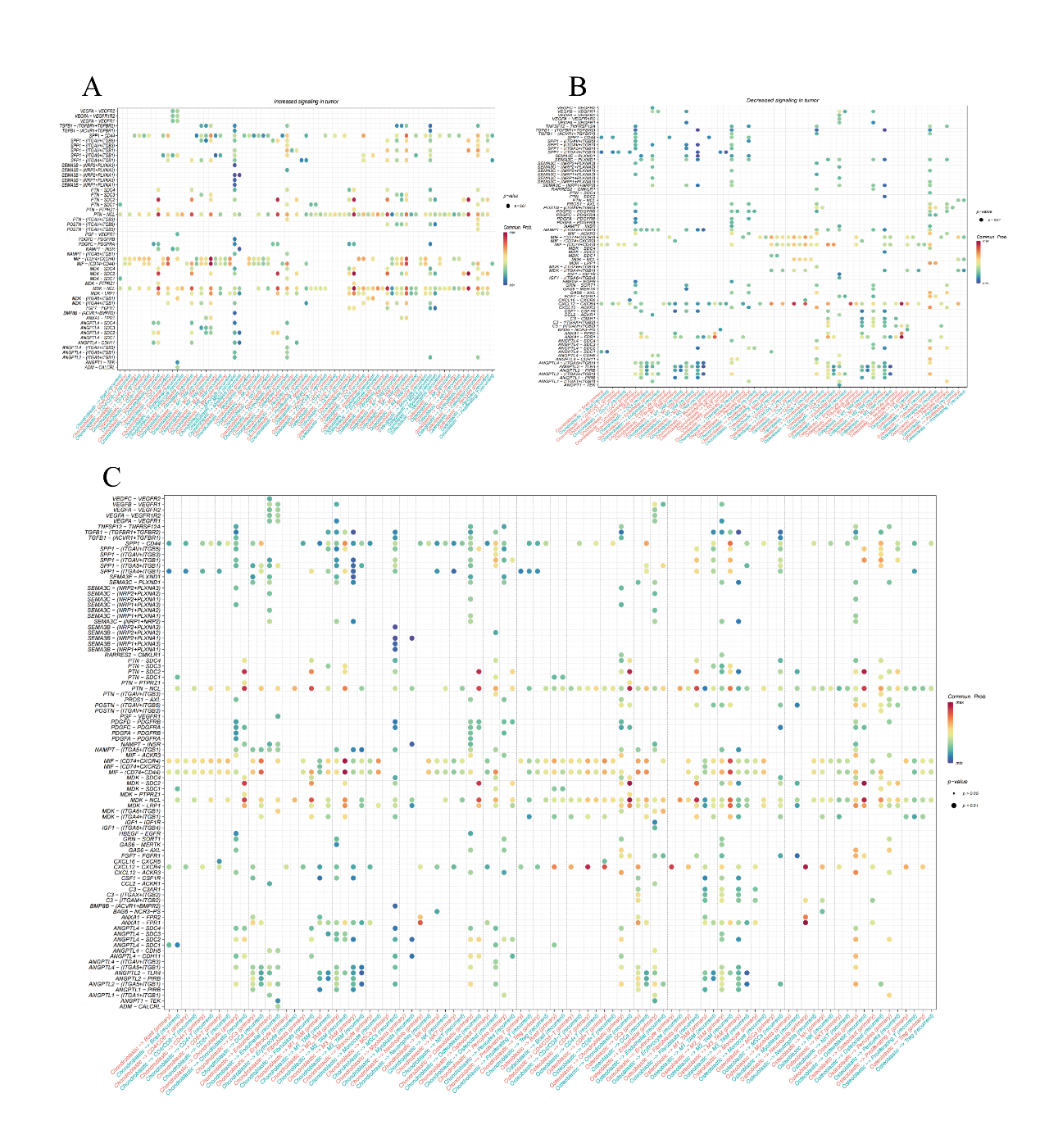


**Figure S7. Probability Analysis of Ligand-Receptor-Mediated Communication in Osteosarcoma Signaling.** (A) Increased signaling in tumor. (B) Decreased signaling in tumor. (C) All communication possibilities.


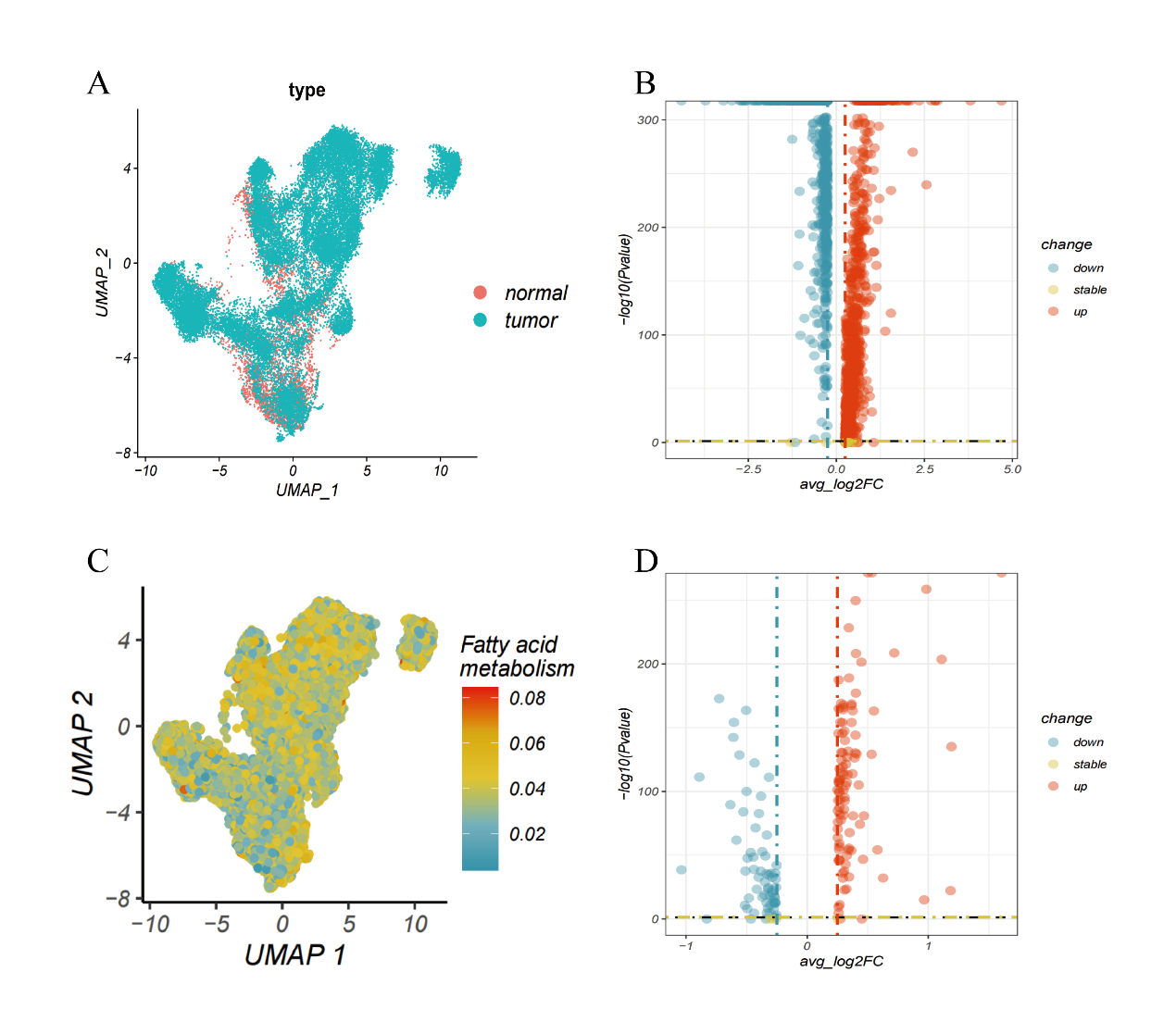


**Figure S8. Differential Gene Expression Analysis.** (A) UMAP plots of osteoblasts from various clinical types. (B) Volcano plots of differential genes between osteosarcoma and normal bone. (C) UMAP plots of individual osteoblasts' fatty acid metabolism. (D) Volcano plots of differential genes between osteoblasts with high and low fatty acid metabolism.


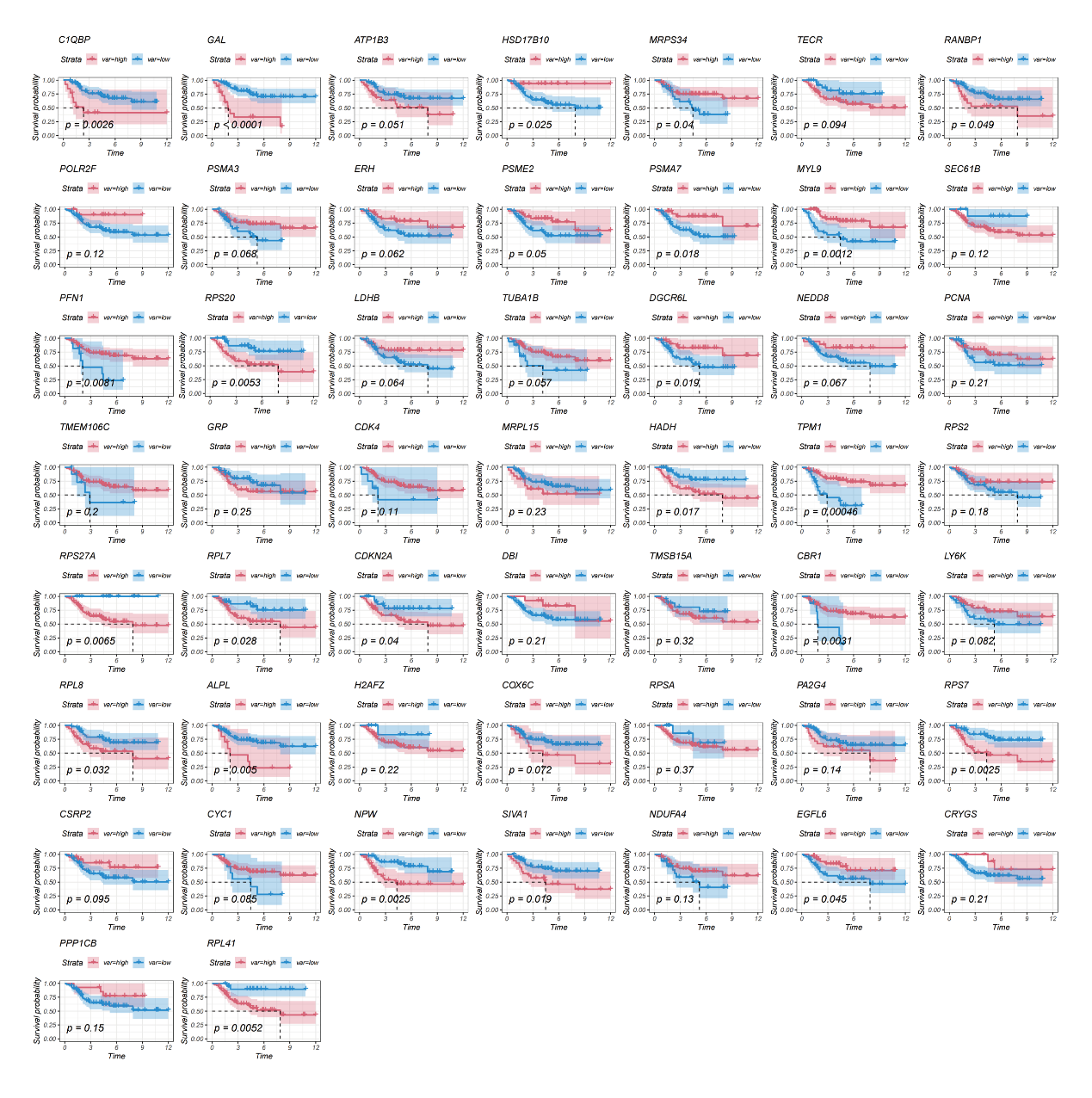


**Figure S9. Validation of OS-FAM's Prognostic Predictive Capacity using the TARGET Database.**


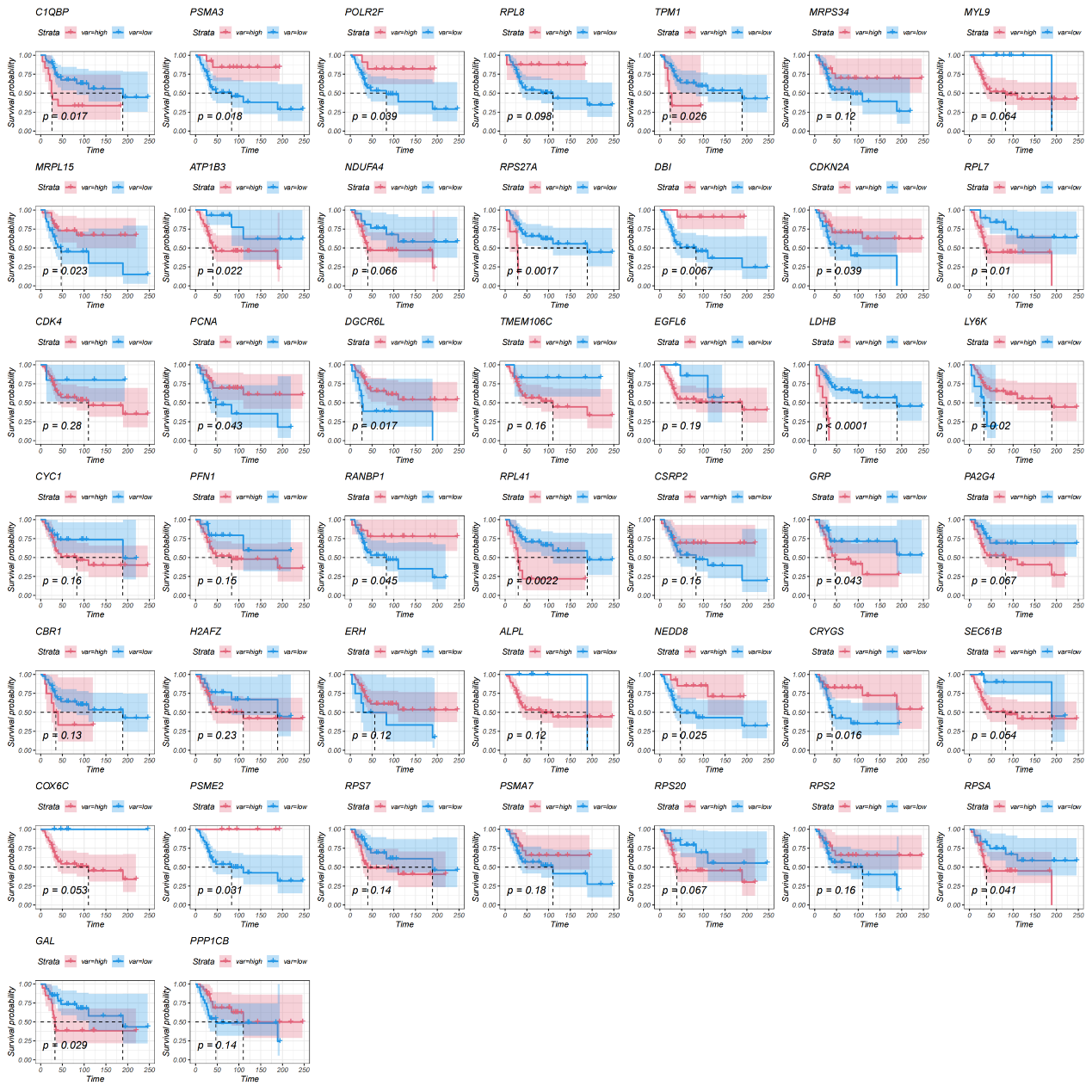


**Figure S10. Validation of OS-FAM's Prognostic Predictive Capacity using the GSE21257 Database.**


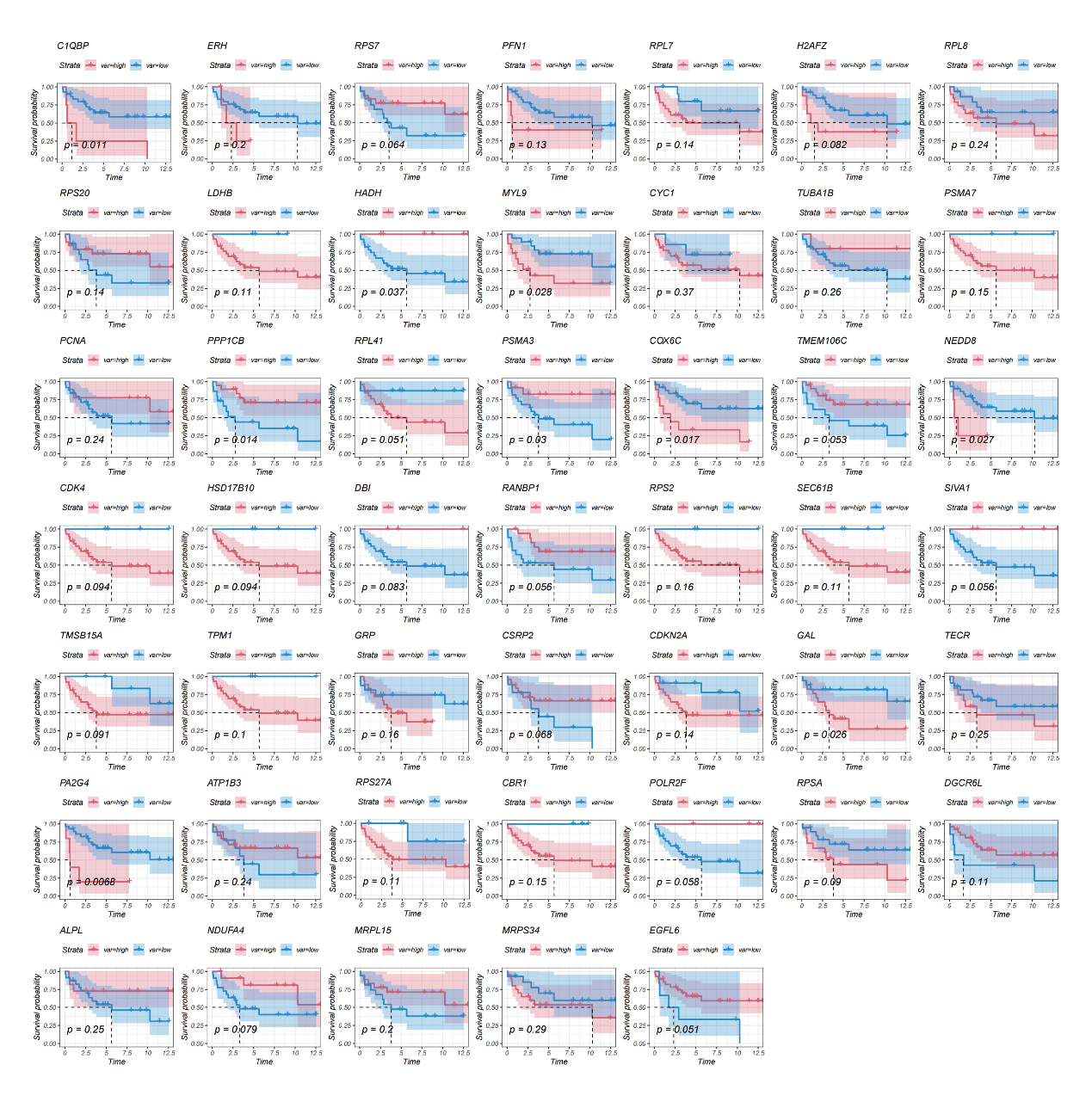


**Figure S11. Validation of OS-FAM's Prognostic Predictive Capacity using the GSE16091 Database.**


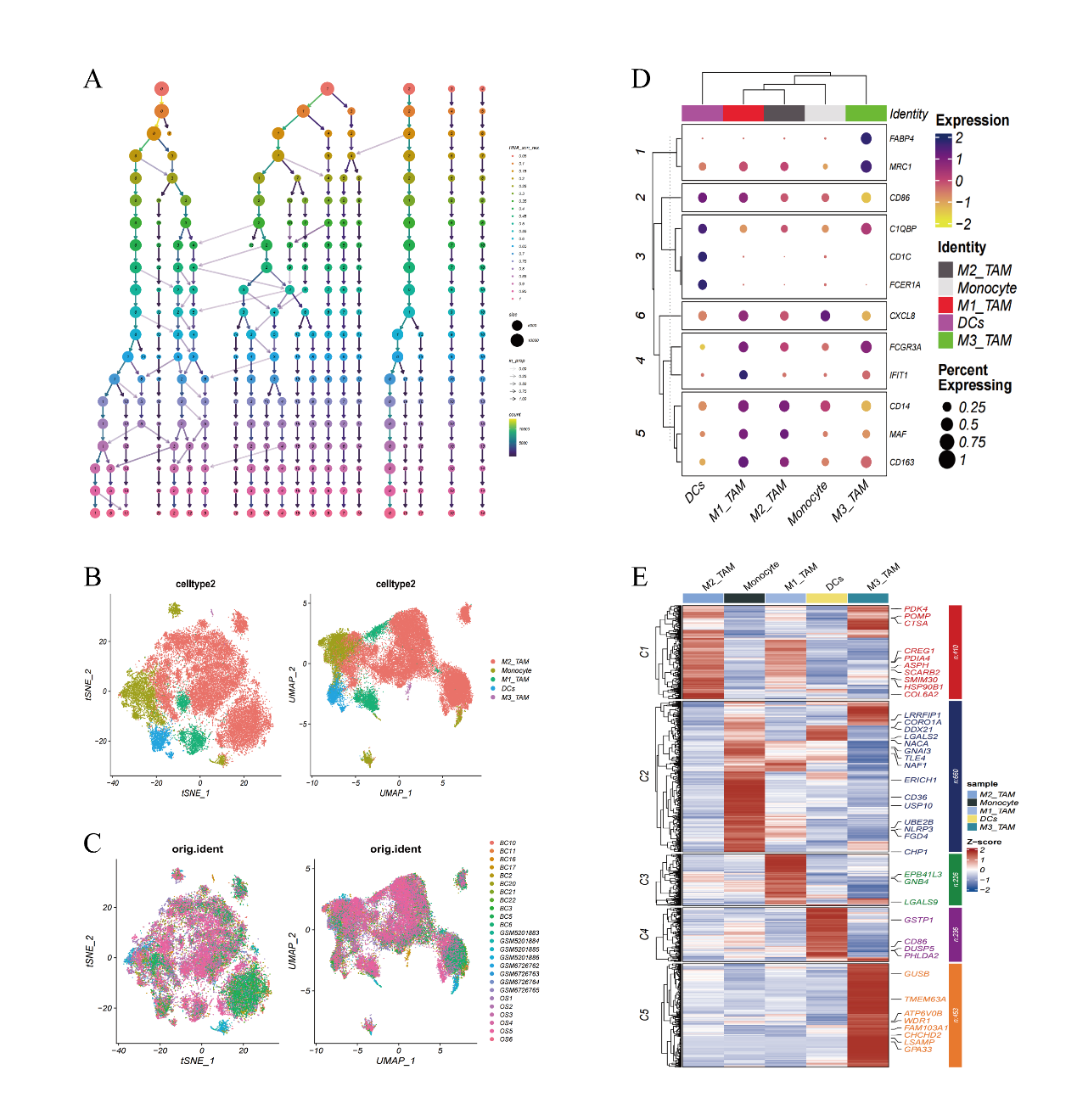


**Figure S12. Dimensionality Reduction, Clustering, Annotation, and Analysis of Mononuclear Phagocyte System Subpopulations.** (A) Clustering dendrogram across resolutions. (B) tSNE and UMAP plots of cell types. (C) tSNE and UMAP plots of different samples. (D) Marker gene displays. (F) Heatmap of DEGs within the mononuclear phagocyte system's five subclusters.

**
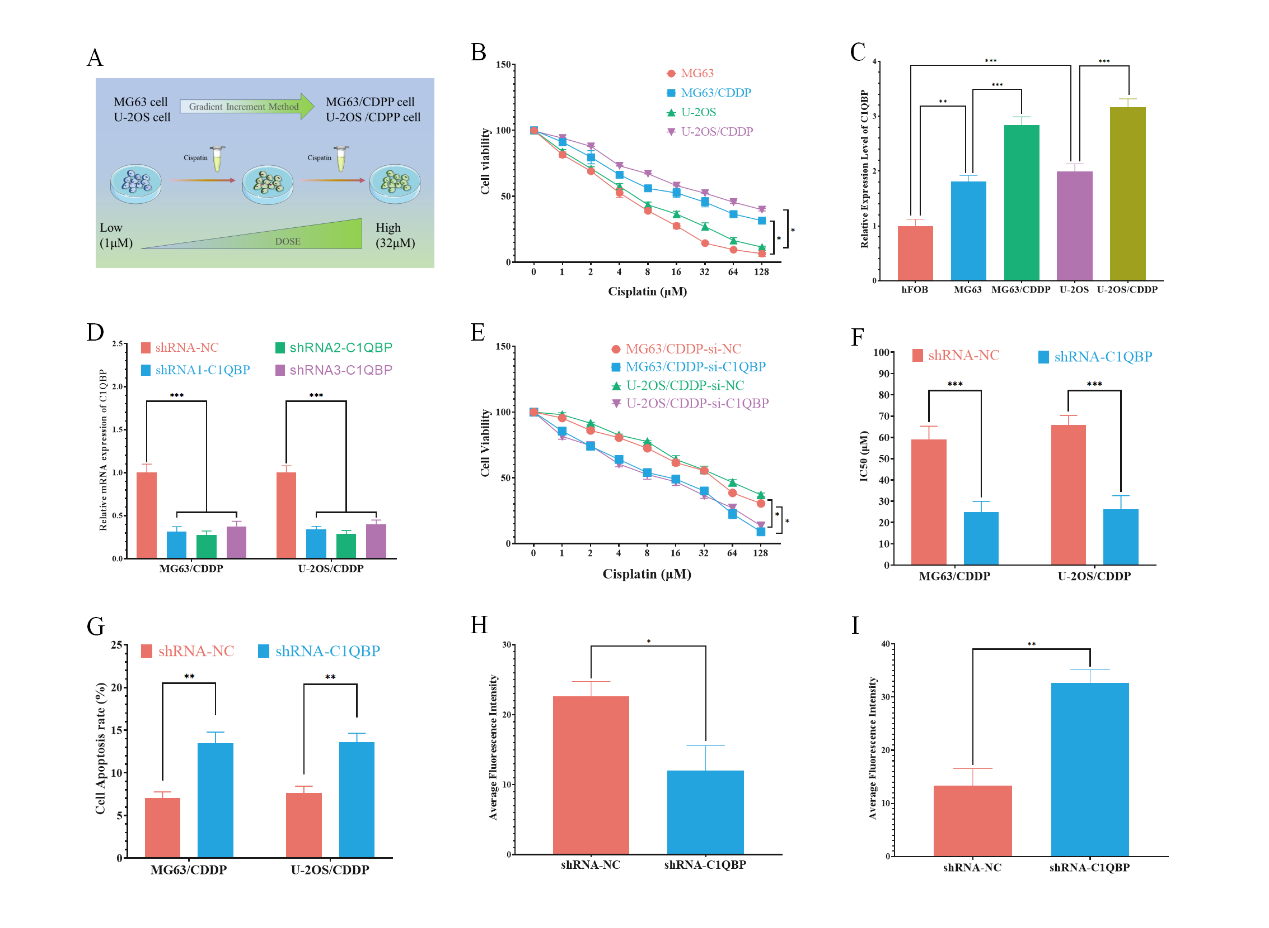
**

**Figure S13.** (A) Establishment Steps for Osteosarcoma Drug-Resistant Cell Lines. (B) CCK-8 Assay Comparing Osteosarcoma Drug-Resistant and Parental Cell Lines. (C) Semi-quantitative Analysis of Western Blot Bands via Image J. (D) qRT-PCR Analysis of si-C1QBP Treatment Efficiency. (E) CCK-8 Method Determination of Cell Viability across Various Cisplatin Concentrations. (F) CCK-8 Method Determination of IC50 Values. (G) Quantitative analysis of apoptosis in osteosarcoma cells. (H-I) Semi-quantitative Analysis of Immunofluorescence from Figures 12D and 12F via Image J. * P < 0.05, ** P < 0.01, *** P < 0.001. (Data mean ± SD, n=3).

**Supplementary Tables**

**Table S1. Primers used for RT-qPCR**

| **shRNA** | **Primer sequences** |
| --- | --- |
| shRNA1-C1QBP | GCACCAGGAGTACATTACTTT |
| shRNA2-C1QBP | CCTTGGACTGGGCCTTATATG |
| shRNA3-C1QBP | TCTGAATGGAAGGATACTAAT |

**Table S2. Primer Sequences of shRNA**

| **Genes（Human）** | **Accession no.** | **Forword (5’-3’)** | **Reverse (5’-3’)** | **Product size (base pairs)** |
| --- | --- | --- | --- | --- |
| C1QBP | NM_001212 | AGAAGCGAAATTAGTGCGGAA | CCACGAAATTGGGAGTTGATGTC | 161 |
| CD86 | NM_001024630.4 | CTGCTCATCTATACACGGTTACC | GGAAACGTCGTACAGTTCTGTG | 133 |
| INOS | NM_000625 | AGGGACAAGCCTACCCCTC | CTCATCTCCCGTCAGTTGGT | 168 |
| CD206 | NM_002438 | GGGTTGCTATCACTCTCTATGC | TTTCTTGTCTGTTGCCGTAGTT | 126 |
| ARG1 | NM_000045 | TGGACAGACTAGGAATTGGCA | CCAGTCCGTCAACATCAAAACT | 102 |
| FABP4 | NM_001442 | ACTGGGCCAGGAATTTGACG | CTCGTGGAAGTGACGCCTT | 183 |
| GAPDH | NM_005430.4 | GAAGGTGAAGGTCGGAGTCAACG | TGCCATGGGTGGAATCATATTGG | 129 |
